# Challenges and Opportunities for Academic Parents During COVID-19

**DOI:** 10.3389/fpsyg.2021.645734

**Published:** 2021-08-18

**Authors:** Eva O. L. Lantsoght, Yvonne Tse Crepaldi, Silvia G. Tavares, Kathleen Leemans, E. W. Misty Paig-Tran

**Affiliations:** ^1^Politecnico, Universidad San Francisco de Quito, Quito, Ecuador; ^2^Engineering Structures, Faculty of Civil Engineering and Geosciences, Delft University of Technology, Delft, Netherlands; ^3^School of Social Sciences, Nanyang Technological University, Singapore, Singapore; ^4^School of Law and Society, University of the Sunshine Coast, Sippy Downs, QLD, Australia; ^5^Translational Radiation Oncology, Physics and Supportive Care, Faculty of Medicine, Vrije Universiteit Brussel, Brussels, Belgium; ^6^Department of Biological Sciences, California State University Fullerton, Fullerton, CA, United States

**Keywords:** academia, academic leadership, childcare, COVID-19 relief, qualitative analysis, quantitative analysis, survey

## Abstract

Parents in academic careers face notable challenges that may go unrecognized by university management and/or policy makers. The COVID-19 pandemic has shed light on some of these challenges, as academic parents shifted to working from home while simultaneously caring for children. On the other hand, many parents found that the shift to working from home offered new opportunities such as working more flexible hours, development of digital skillsets, and increased involvement in the education of their children. In this article we explore the work-related challenges and opportunities experienced by academic parents as a result of the COVID-19 pandemic and offer potential long-term solutions for academic parents and their universities. We use the following methods: (1) a literature review focused on identifying the work-related challenges academic parents faced prior to the pandemic, as well as the impact of the pandemic on scientists and working parents and (2) administer a world-wide survey with the goal of identifying the challenges and opportunities associated with parenting and academic work through the COVID-19 lockdown (304 total responses; 113 complete). Moving forward these findings have enabled conclusions to be drawn in order to shape a new normal. Our aim is to offer university administrators, policy makers, and community service providers with ways to provide additional support for academic parents as well as provide tools for academic parents to learn successful strategies directly from their peers.

## Introduction

The COVID-19 pandemic required academics to work from home and switch to blended and hybrid teaching, requiring a quick shift in teaching materials and style. Simultaneously, school closures and the loss of other forms of childcare required a greater contribution of parents toward both the education and care of their children. The COVID-19 pandemic and related lockdown(s) created increased challenges for academic parents. In particular, academic parents experienced intensified workloads associated with shifting work from in-person to virtual platforms in conjunction with expanded childcare and/or homeschooling. Short-term solutions transitioned to long-term obstacles as parents began planning for extended periods with reduced or no childcare. A US survey conducted in May 2020 (National Coalition for Public School Options, 2020) found that 21% of parents would not be comfortable sending their children back to an in-person learning facility in September 2020. In addition, the number of after-school programs for children's outdoor environmental and science education had decreased because of the pandemic. Sixty-three percent of these groups remain uncertain about their ability to ever reopen (University of California - Berkeley, 2020). Working parents depend on such after-school programs as a form of childcare and the closure of these programs may have long-lasting effects. On the other hand, academic parents found opportunities in working flexible hours, developing skills related to and taking full advantage of new digital tools and cloud environments, and increased involvement in the education of their children.

The sudden onset of lockdowns associated with the COVID-19 pandemic has reshaped work-life balance for academic parents globally, in effect, creating both additional challenges and unique opportunities for maintaining work productivity. At the same time, parents had to adjust to additional time pressures associated with increased childcare duties. In this study we aim to identify these challenges and explore the opportunities identified by individuals, as well as support at the university level, with a focus on long-term solutions. We use the following methods: (1) conduct a literature review on the general work-related challenges academic parents face, and present results on the impact of COVID-19 to working parents and academics, and (2) administer a worldwide survey to investigate the impacts of the COVID-19 pandemic to academic parents in order to explore the challenges and opportunities presented by the pandemic. We combined multiple-choice (Likert-scale) and open-ended questions to allow for mixing quantitative and qualitative methods, which gives us more in-depth insights into the effects of the pandemic on academic parents. This study is important for both academic administrators and academic parents. For the former, it provides insight into the lived experiences of academic parents and helps to identify additional types of support that may prove effective for reducing stress and sustainable career growth. For the latter, it provides a tool to reflect on their situation, learn from lived experiences of others, use these insights to adapt to the new normal, and gives them data for necessary discussions with their administrators.

## Literature Review

### Challenges and Opportunities for Academic Parents Prior to The COVID-19 Pandemic

#### Impact of Having Children on Academic Careers

To develop the survey used in this study, we reviewed literature on academic parents and identified recurring themes emerging from this literature. Literature on academic parents prior to COVID-19 focused mostly on academic mothers in the Anglo-Saxon countries, with few studies from other regions such as Germany (Bomert and Leinfellner, 2017), Iceland (Rafnsdóttir and Heijstra, 2013), and Asia (Lau, 2020). Child-bearing women academics are disproportionally represented throughout the literature, partially because there is evidence showing negative impacts on achieving tenure (Harris et al., 2019) related to having children; whereas men's careers are boosted by having children (Windsor and Crawford, 2020). Academic mothers are subject to career delays due to pregnancy, childbirth and nursing, and many university campuses are ill-equipped to accommodate the physical aspects of motherhood (Trussell, 2015; McCutcheon and Morrison, 2018; Mirick and Wladkowski, 2018). This is perhaps a larger reflection of how academia views motherhood (Mirick and Wladkowski, 2018; Moreau and Robertson, 2019), which factored into the decision by some academic women to remain single and childless throughout their career (Mason et al., 2013). Women faculty are more likely to be partnered with someone working full-time (Bascom-Slack, 2011) while male faculty are less likely to share the burden of unpaid household and childrearing work with their partners. Culturally, “‘*intensive mothering’ has become the dominant cultural script in the West*”: mothers are expected to show devotion and self-sacrifice (Moreau and Kerner, 2015). This focus on mothers has been broadened to all parents in recent years as traditional childcare roles are shifting and fathers play a more active role in childrearing as the cultural perception of what it means to be a “good father” is changing. Academic fathers who take on care and household responsibilities are also pressed for time between family and work responsibilities (Wilton and Ross, 2017; Derrick et al., 2019; Harris et al., 2019; van Engen et al., 2019).

#### Identified Themes

We identified three recurring themes throughout our initial literature review related to the challenges experienced by academic parents. These emergent themes shaped the format of our survey including the structure and topics of our questions. The themes include:

*Theme 1: Childcare:* Childcare coverage generally implies that academic parents have to leave their office at a fixed time. To find extra hours for completing work, many academic parents report working early mornings and late into the evenings (Weeks, 2018), often when their children are sleeping. When regular childcare arrangements fall through, for example when children get sick or during snow days, academic parents experience the immense difficulty of having tight schedules disrupted (Bascom-Slack, 2011).

*Theme 2: Work-Life Balance:* Balancing the demands of an academic career while simultaneously raising a family are sources of potential conflict for academic parents as they often find themselves unable to meet all these demands (Harris et al., 2019; van Engen et al., 2019). Academic careers are often associated with intense work pressure, heavy workloads [50–60 work hours per week (Poronsky et al., 2012), and meetings scheduled during non-traditional work hours - at night and over the weekend (Bomert and Leinfellner, 2017)]. In addition, sedentary lifestyles (Michailidis, 2008) and sleep-deprivation are often experienced by academic parents (McCutcheon and Morrison, 2018). As such, negative coping strategies are often employed to alleviate the stress and guilt including: smoking, drinking, and overeating (Poronsky et al., 2012; Wilton and Ross, 2017). On the flip side, academic parents often benefit from increased opportunities for flexible schedules, flexibility in terms of tasks they take on, and possible career paths (research-, teaching-, or admin-oriented career) (Bomert and Leinfellner, 2017; Wilton and Ross, 2017; Windsor and Crawford, 2020). Increased flexibility also comes with the potential for undefined or irregular work times, which make it increasingly difficult to define dedicated work and family time (Rafnsdóttir and Heijstra, 2013). On a positive note, we learned that for some scholars, becoming a parent means adding a layer of purpose to their work, where they feel their research contributes to creating a better world for their children (Comer and Stites-Doe, 2006; Trussell, 2015).

Recommendations from academic parents for balancing academic work and family include: (1) dedicating non-teaching days to service to the profession and university or writing and protecting that time, which helps with setting boundaries for when to work, what work can be done, and reduces the uncertainty of when these tasks can be handled (Windsor and Crawford, 2020), (2) establishing what is “good enough” for oneself, which also helps with setting boundaries for work (Moreau and Kerner, 2015), (3) negotiating with administrators, when necessary, for example when family responsibilities temporarily demand more time of the academic parent (Bascom-Slack, 2011; Windsor and Crawford, 2020), (4) identifying one's priorities and making choices for work and family (Weeks, 2018; Windsor and Crawford, 2020), and (5) seeking mentors within the university administration and in their personal lives who can advise and guide academic parents, and developing healthy social support networks so that the academic parents can have nourishing relationships outside of work and family (Michailidis, 2008; Isgro and Castañeda, 2015; Trussell, 2015; Hertlein et al., 2018).

*Theme 3: University Support*. The final theme deals with the topic of university support and university policies that support (or hinder) academic parents. Most of the existing literature focuses on individual experiences of academics including struggles, work-life balance, and coping mechanisms. University support and policies are discussed less frequently. When these topics are discussed, it is mostly in the light of career advancement and, specifically, tenure and promotion requirements. For example, parents using parental leave or part-time work are often held to the same standards in their evaluations as full-time academics (Klocker and Drozdzewski, 2012; Derrick et al., 2019; Huppatz et al., 2019; van Engen et al., 2019). The other broader topic regarding university policies that is discussed in the literature is the idea of moving toward a “culture of care” in universities. This philosophy includes noticing, connecting, and responding to the various needs of people working and studying on campus (Springer et al., 2009; Moreau and Kerner, 2015; Mirick and Wladkowski, 2018; Wladkowski and Mirick, 2019).

### Impact of COVID-19 on Working Parents and Academics

A new emerging theme, which informed a block of questions in the survey, focused on the impact of COVID-19 on academic work. Several recent studies have addressed either its impact on working parents or on academics, and in one recent case the authors also studied parenting roles and the impact on time use (Deryugina et al., 2021).

Several studies have focused on the influence of the pandemic and lockdown on scientific output. An analysis of publication outputs early in the pandemic (between March and April 2020) showed that women's publishing rates did not increase proportionally to men's (Viglione, 2020). Squazzoni et al. (2020) analyzed both manuscript submissions and peer review requests through the data of publisher Elsevier and found that women accepted fewer invitations for peer review. In addition, women at later career stages experienced the largest negative impact on the rate of manuscript submissions as a result of the pandemic and lockdown. A study published in May 2020 by Pinho-Gomes et al. (2020) showed similar impacts to women as last authors (senior authors) of journal articles. Most purely bibliometric analyses focused on data between March and April 2020, though it is of note that when data from June through November are included, a partial recovery in publication rates is observed. The authors attributed the initial drop in publications by female researchers to increased care responsibilities for children and relatives, as well as to the larger teaching and service loads most female scholars take on. The increased workload caused by the shift to online teaching thus disproportionately affected women (Kramer, 2020), leaving less time for research and the development of publications.

A large-scale (25,307 respondents) international study (152 countries) (Rijs and Fenter, 2020) focused on the overall impact of the pandemic on scientists, and found that 70% of the respondents managed to perform the majority of their work tasks, 10% mentioned their work was not affected at all, and most researchers (72%) were positive about the support they received from their university. It is important to note that there was a strong geographical influence, with the largest negative impact on scientists' work occurring in South America and the fewest impacts occurring in South Korea and Switzerland, possibly related to different local lockdown measures, their duration, and the universities' responses to these measures. This study did not address the influence of gender and/or care-giving roles.

A recent study (Deryugina et al., 2021) focused on the time use (in the categories research, other work, commute, childcare, housework, sleep, and other) on a daily basis of 19,905 academics using a time-use survey. In separating out the data by gender, and looking at parenting roles, they found that all academic parents reported a reduction in time spent on research per day. This reduction of time spent on research is greater for academic mothers than for fathers: academic mothers lose about an hour a day on research time, whereas academic fathers lose about 30 min of research time as compared to academics without parenting roles, and the difference is largest for academic parents with one child. For academic parents with more children, the authors observe that academic fathers lost increasingly more research time, and that the gap with academic mothers became smaller. The greatest impacts were experienced by academic parents with children under the age of seven. The authors concluded that the greatest adverse productivity effects of the pandemic were experienced by female academics with young children. In addition, Gewin (2021) reported findings with regard to stress and burnout from a survey of 1122 US faculty members, which showed that 70% of faculty members reported feeling stressed in 2020 as compared to 32% in 2019. The impact was higher on women faculty, with 75% of women feeling stressed as compared to 59% of men.

A second set of studies examined the impact of COVID-19 on working parents from multiple disciplines. Working parents in general spent more hours per week on housework and childcare/homeschooling as a result of the lockdown during the COVID-19 pandemic (Benzeval et al., 2020; Lee, 2020; Reisz, 2020). The combination of work and parenting during lockdown universally increased stress levels for parents (Keong, 2020; Keong et al., 2020a,b; Lee, 2020), a result of which led to an upward trend in the incidence of yelling, screaming, child abuse, and marital distress. Additionally, parents worried about the physical and mental health of their children, falling behind academically, children's loneliness and lack of social interactions, screen time and online safety, and their own performance as parents in uncertain times (Jones, 2002). A few studies (Keong, 2020; Lee, 2020) outlined coping mechanisms that have proven effective for working parents during COVID-19 including: (1) giving children daily challenges to keep boredom at bay, (2) enjoying the increased amount of quality family time, (3) more equitably sharing in responsibilities between both parents, and (4) rethinking parenting altogether, with a focus on a “*simple and quiet*” life (Lee, 2020) instead of busy schedules with many activities. As solutions for the increased parental stress, Keong et al. (2020a) recommend the use of online marital counseling and self-directed parenting interventions, such as Parent-Child Interaction Therapy and Triple P (based on 5 core positive parenting principles).

### Research Gap

Recent research associated with the pandemic tended to focus specifically on marital stress and the combination of work and homeschooling for working parents in general, or on the reduction in publications by academic women. This study addresses the effect of the COVID-19 pandemic on academic parents more broadly and holistically - exploring the challenges experienced between genders and across regions, ethnicities, and academic ranking. Analyzing the responses for these various categories addresses a gap in the current literature and sets our study apart from previous work. The international character of the study adds to the current body of knowledge by examining the perspective of academic parents world-wide and in regions where lockdown measures were significantly different. The study aims to identify the sources of challenges academic parents face during COVID-19. In addition, we aim to articulate new opportunities. This research addresses the effects of the COVID-19 pandemic on academic parents by quantitatively exploring two aspects of performance—research and teaching—and by qualitatively exploring the impact on academic work by allowing academic parents to expand on their individual experiences. The combination of quantitative and qualitative methods allows for a deeper understanding of the particular challenges and opportunities that academic parents face. The international context of the work gives insight into different types of policies and university support that have been provided, and how these policies (or lack thereof) have been received by academic parents.

## Methodology

### Research Design: Survey and Participants

Based on our initial literature review on academic parents, we identified three main themes to study: (1) the effect of childcare changes due to the COVID-19 pandemic on academic parents, (2) the influence of the pandemic on time usage and work-life balance experienced by academic parents, and (3) the level of university support received during the pandemic. In addition, based on the literature review on the impact of COVID-19 on working parents and academics, we included a block of questions to evaluate the effect of parenting through the pandemic on work (research and teaching).

We found two approaches for methodology in the literature. The work on the impact of COVID-19 on the publications of academics is purely quantitative work, focusing on bibliometric data. However, most references related to academic parents/mothers focus on qualitative approaches: both autoethnographies and interviews of which the data were then used for inductive thematic analysis or grounded theory. We performed a hybrid approach incorporating both quantitative and qualitative data.

We investigated the themes identified from the literature with a worldwide online survey (IRB approval nr 2020-056M obtained through Universidad San Francisco de Quito), which accepted responses between Oct 17th and Nov 5th of 2020. The four main blocks of questions were based on the four identified themes, in addition to an initial block of questions to identify the socio-demographic characteristics of the respondents. The survey contained open-ended questions, closed-ended multiple-choice questions, sliding-scale type questions (Likert scale), and self-reported time logs. In terms of the psychometric characteristics of the survey, we leaned on the literature to draft the survey, and then discussed improvements in the questions and topics to explore within our multidisciplinary research team to optimize construct validity. No further internal consistency checks have been carried out, as the aim of the survey is not to develop a standardized instrument but to understand the impact of the pandemic on academic parents. The survey contained 41 questions and is presented entirely in the Supplementary Material.

The survey was open to all academic parents, starting at the level of Ph.D. candidate. We used the academic ranking system from the USA^1^ for self-identification of the position of the participants, using assistant, associate, and full professor as the academic ranks in the promotion system, and lecturer^2^ and researcher for those who have an appointment that is specific for lecturing or research, but that does not span both. Participants were recruited through social media and via our email contacts, and as such represent a self-selecting convenience sample (Huppatz et al., 2019). This approach is sufficient for the purpose of this research, which aims to understand the lived experiences of academic parents during the pandemic.

The data collection was impacted by COVID-19. In-person interviews were not an option, and after debating the possibility of taking traditional interviews per video conference, we opted against this approach, as this would require a larger time investment of the participants than what they possibly had available. Using the online survey had two advantages: (1) it allowed us to reach regions outside of our direct network, thanks to social media, and (2) it allowed academic parents to participate at their own convenience, and to return to the questionnaire at their convenience, potentially after interruptions.

### Data Analysis

#### Quantitative Data Analysis

After excluding the participants who had not provided or who had withdrawn informed consent, we obtained the dataset of 304 responses. We added respondent codes (RXXX with XXX the associated number) to the dataset to have a unique and anonymous identifier for each respondent (Allen and Wiles, 2016), and provided this dataset in the public domain. In the full dataset, there is one set of responses completed only up to the first block of questions regarding childcare (hereafter, “partially complete surveys” =177), and another set completed until the final question (hereafter, “completed surveys” = 113). We then developed the report of the responses to each question separately on the survey platform, and read the responses (each response to all questions individually, as well as all responses to each question separately) to get an initial handle on the scope of the results. We then used the filtered dataset to analyze the data in self-developed spreadsheets by breaking down the survey responses of individual questions per socio-demographics category (19 analyses). We also cross-checked these outcomes with breakdowns set up in the survey platform. In addition, we used the Qualtrics survey platform software to generate cross-tabulations between the questions, resulting in 39 crosstabs, which we further analyzed in MS Excel. To study a possible relation between two variables, we calculated percentages for each category (e.g.,. % males that answered Strongly agree, etc.) and then used a Kruskal-Wallis test in SPSS V16 based on those percentages for each classification (e.g., childcare situation, support, and work-life balance). The Kruskal-Wallis test, a non-parametric method, suitable for ordinal data, was selected because of the small sample size and deviation from normality to test if the results in different categories originate from the same distribution (Dunn, 1964; Conover and Iman, 1979; Jamieson, 2004). The confidence level was set at 95% and a *p*-value of <0.05 indicated a strong correlation and a value of 0.1 > *p* > 0.05 was considered to indicate a weak correlation. We developed a separate spreadsheet for the analysis of the time logs.

#### Qualitative Data Analysis

The survey design was informed by the literature review, and the qualitative thematic analysis was based on the themes emerging from the textual responses to individual open-ended questions. The qualitative analysis was carried out by using the principles of inductive thematic analysis (Patton, 1990; Braun and Clarke, 2006) per identified topic and was based on four main steps: (1) coding; (2) code filtering; (3) emerging themes; and (4) questions summary.

In the coding stage (step 1), all responses were read in detail, and emerging codes were identified and tagged on the Qualtrics survey platform. Each code was tagged in each response—where relevant—only once as it refers to content and not the citation of any specific words. At the same time, a “*memoing*” document was created (Lofland et al., 2006) with the description of all codes to ensure consistency throughout the analysis and to avoid repetition of the meaning of each code. Each question had its own set of codes and *memoing* document. Once all responses for each question were coded, the codes were reassessed and filtered (step 2) along with their memos to avoid repetition and to reframe the wording for consistency where needed. After reassessing and filtering all codes, a spreadsheet was created for each question and all codes listed. The number of mentions for each code was also added to these tables and, at this stage, the overarching themes for each question were identified (step 3). These themes were not predetermined; they emerged from the responses and the mentions for each code under these overarching themes. Within each overarching theme the codes were listed from the most frequently mentioned to the least mentioned (see example in Annex A). Finally, a summary was written for each question (step 4). These summaries reported on the meanings behind those numbers, i.e., how the themes and codes relate to each other to “tell a story” of the reported experiences of academic parents during COVID-19. The mention numbers give us an insight in how often similar responses were given. These summaries were structured based on the identified overarching themes and were illustrated with quotes referring to the codes within those themes. To do so, all responses under those codes were revisited in Qualtrics and representative quotes could then be extracted to illustrate the key themes. The most significant “trends” —the overarching themes—were reported in the results along with the number of mentions and specific quotes were used to illustrate where relevant.

## Results and Analysis

### Survey Responses and Demography

In total, our survey received 304 responses (with consent). Of these, 113 were considered complete surveys (i.e., respondents reached the final question), with between 105 and 113 respondents answering each question from the quantitative portion and between 69 and 113 respondents answering the open-ended questions. 177 partially complete surveys (i.e., respondents reached the end of the block with questions about childcare) were analyzed as well (note that the 113 complete surveys are a subset of the 177 partially complete surveys). Respondents were allowed to answer what they preferred, and could return to the questionnaire at their own convenience when using the same IP address. The time spent on the completed surveys ranged between 8.7 min and 142 h (the median across the 113 respondents was 23.6 min), where the longer times indicated that participants returned to an unfinished survey.

Table 1 shows that the majority of participants with complete responses (*n* = 113) were white (71%), and almost two-third self-identified as women (63%). We did not receive responses from academics self-identifying as Indigenous or First Nations and we had one Black or African American respondent; as such, some ethnic groups were not well-represented. More than half (59%) of the participants were aged between 35 and 44 years old. Most worked as lecturers (21%), assistant professors (21%), or associate professors (26%) at their university. Ninety-three percent of respondents were living together with a partner and the majority of respondents had one or two children (mean 1.4). Almost two-thirds of their children fell between the ages of 3 and 12 years old (64%). The respondents were distributed across all disciplines (31% life sciences, 24% science and engineering, 22% social sciences, 17% humanities, and 6% multidisciplinary).

**Table 1:** Socio-demographics of participants of completed surveys, *n* = 113.

	**Total**	**Ph.D. candidate**	**Researcher**	**Lecturer**	**Post-doctoral researcher**	**Assistant professor**	**Associate professor**	**Full professor**	**Other appointment**
	***n* = 113**	***n* = 12**	***n* = 4**	***n* = 11**	***n* = 8**	***n* = 24**	***n* = 29**	***n* = 12**	***n* = 13**
**Age**									
25–34	18%	58%	25%	9%	29%	8%	7%	8%	40%
35–44	59%	33%	25%	45%	71%	88%	55%	58%	50%
45+	23%	8%	50%	45%	0%	4%	38%	33%	10%
**Gender**									
Male	36%	25%	50%	9%	38%	50%	34%	42%	40%
Female	63%	75%	50%	91%	63%	50%	66%	50%	50%
Other	2%	0%	0%	0%	0%	0%	0%	8%	10%
**Race**									
White	71%	75%	50%	64%	71%	75%	76%	50%	70%
Asian	14%	17%	0%	27%	14%	13%	14%	8%	20%
Latino/Hispanic	8%	8%	25%	0%	0%	8%	3%	33%	0%
Mixed	4%	0%	0%	9%	0%	4%	3%	8%	10%
Other	3%	0%	25%	0%	14%	0%	3%	0%	0%
**Relationship status**									
Living together	93%	92%	75%	91%	100%	100%	97%	92%	73%
Single	5%	8%	25%	9%	0%	0%	0%	8%	18%
**Age of children**	(*n* = 154)								
0–1 year	6%	6%	0%	0%	8%	6%	7%	7%	0%
1–2 years	17%	6%	40%	7%	23%	28%	5%	7%	25%
3–6 years	32%	50%	20%	29%	38%	38%	37%	7%	25%
7–12 years	32%	28%	20%	43%	23%	22%	37%	53%	50%
13–18 years	13%	11%	20%	21%	8%	6%	15%	27%	0%

### Support and Infrastructure

#### University Support

The quantitative analysis of completed surveys showed that academic parents had varying perceptions toward their university support (Table 2). In general, parents felt understood by their colleagues and supervisor, but the infrastructure to provide tangible support was often lacking. The respondents perceived Universities as not actively engaging in measures (e.g., actively helping find childcare alternatives) that directly supported achieving the critical balance between work and parenting responsibilities. Respondents had mixed views on whether university policies were “welcoming” toward academic parents; the responses were almost evenly distributed between disagreeing, neither agreeing nor disagreeing, and agreeing. Academic parents generally felt that their colleagues (49% somewhat or strongly agree) and supervisors (55% somewhat or strongly agree) understand the challenges of being an academic parent.

**Table 2:** Breakdown of responses to questions related to university support.

**Statement**	***N***	**Strongly disagree**	**Somewhat disagree**	**Neither agree nor disagree**	**Somewhat agree**	**Strongly agree**
I feel supported by my university as an academic parent during the pandemic	113	23%	20%	22%	22%	12%
My university has actively helped me achieving balance between work and childcare duties during the pandemic	112	38%	25%	23%	9%	4%
My university has actively helped me find childcare alternatives	112	64%	13%	19%	4%	0%
My university has a welcoming attitude to parents	112	15%	19%	32%	25%	9%
My colleagues understand the challenges of being an academic parent	113	15%	19%	17%	34%	15%
My superiors understand the challenges of being an academic parent	113	14%	14%	17%	36%	19%

The perception of university support differed depending on the age of the child(ren) of the academic parents: 44% of parents of infants strongly disagreed with “I feel supported by my university as an academic parent during the pandemic,” vs. 19% of parents of toddlers, 32% of parents of preschoolers, 18% for parents of primary school-aged children, and 15% for parents of secondary school-aged children, as also expressed by R232 (parent of 2 preschoolers): “*feel disconnected with peers and teachers, do not know updates from institution.”*

The qualitative analysis for open-ended questions regarding university support provides further insight into the types of support universities have (or have not) provided, and how this support helped (or hindered) academic parents. About half of the respondents mentioned that their university made changes to workload and flexibility while almost half of the respondents observed that their university has done nothing to provide support. Some universities provided advice and training, arranged leave accommodations and financial support, or made childcare arrangements. Universities have been less successful finding solutions for working parents that are universally well-received and that will last long-term. For example, where universities provided childcare solutions, academic parents reported that these solutions did not serve their needs or that the number of available places was too limited. Even though it was reported that Universities have communicated that they understand the demands facing academic parents with children at home, this understanding has not often resulted in tangible changes to workload or performance requirements. As R012 reported:

*The VC [Vice Chancellor] sent a message at the start of the pandemic saying he understands that parents won't be able to work as much. However, nothing changed at the departmental level, and the workload actually increased due to extra demands on admin and new blended teaching*.

With respect to university support for those on fixed-term contracts, we gathered the following themes: impacts on contract and review procedures for almost half of the respondents, nothing in almost one third of the cases, financial support, and flexibility, with the latter two mentioned only by a small minority of respondents. Almost half of the respondents indicated that this situation did not apply to them. The mentioned impacts on contract and review procedures generally related to issues experienced by all academics (e.g., tenure clock freezes for all faculty or considerations of the impact the lack of research during the pandemic had on research output) with no accommodation specifically related to academic parents, as mentioned by R243:

*I have noticed how productive my colleagues without children have been. I have done no research since the pandemic started, although my teaching hasn't suffered. Unfortunately, this means I won't get a promotion any time soon as there is no allowance for not publishing*.

#### Childcare Arrangements

Using the partially complete responses to understand the impact of childcare arrangements on academic parents during COVID-19, we received between 154 and 177 responses in the quantitative portion and between 122 and 152 responses in the open-ended questions. We have analyzed the partially completed responses for this topic, as the socio-demographics of the respondents represent a more diverse sampling in comparison to the complete responses for the whole questionnaire. Among the respondents to the questions of the childcare situation (*n* = 177), almost three-quarters were white (70%) but there was also a considerable amount of Asian (11%), Latino/Hispanic (9%), mixed (6%), and others. The proportion of women was higher (67%) than for the completed surveys (63%). The majority had a full-time academic appointment. Sixty-nine percent of the respondents (*n* = 100) had a partner who worked full-time (mean 0.89 FTE).

Table 3 shows that more than half (53%) of all academic parents had no assistance with childcare during the initial lockdown and about a quarter (23%) remained so during partial reopening^3^. Comparing the responses for childcare during lockdowns and partial reopening, we note that there was a reduction in childcare resources used per family. Parents used a combination of childcare resources before COVID-19 (e.g., school, plus after-school programs and/or grandparent or nanny); however, the average number of modalities decreased during lockdown and did not fully recover during partial reopening. From the open-ended questions, we note that the responses ranged considerably. For example, a small number of academics expressed (in the open questions) safety and financial concerns when opting against hiring a nanny. Conversely, several, mostly from countries with a tradition of hiring live-in maids or nannies, reported reliance on this childcare solution, while others relied on help from neighbors (e.g., forming “pods” in the United States).

**Table 3:** Source of childcare pre-COVID-19, during initial lockdown and partial reopening.

**Number of entries**	**Pre-COVID**	**Initial lockdown**	**Partial reopening**
	***n* = 310**	***n* = 210**	***n* = 258**
After school care	13%	1%	3%
School	41%	5%	26%
Grandparents	3%	7%	4%
Nanny/Au pair	5%	4%	4%
Daycare	29%	4%	27%
Stay at home partner	2%	18%	9%
Other	2%	10%	2%
No childcare	4%	53%	23%

*Multiple selections per respondent (n = 177) are allowed*.

Besides childcare responsibilities, about half (52%) of the respondents reported that they have additional responsibilities as an informal caregiver, mostly for their partner (21%) and elderly relatives (18%).

#### Schooling

**Figure 1:**
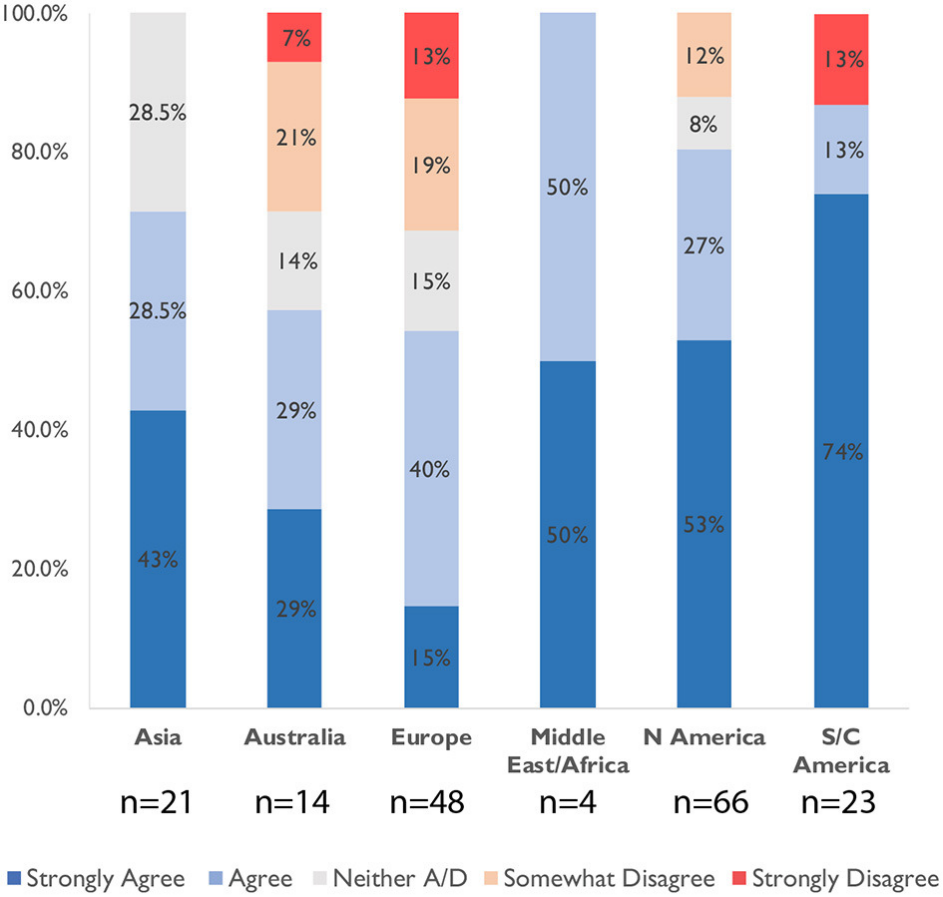
Academic's agreement with the statement “I am worried about sending my children to school or daycare during this pandemic” broken down by region, *n* = 176.

During partial reopening, most young children returned to daycare; however, not all children resumed school in-person, and the availability of after-school care dropped considerably. This may be attributed to schools remaining closed or continuing to offer online education even after reopening, depending on the region. Academic parents who have children back in school or daycare stated that the unpredictability of the situation was a source of worry as mentioned by R245: “*Never know when a case will shut down school or daycare*.” Parents worried about sending children back to school (Figure 1), more so in the Americas (especially Central and South America) and the Middle East/Africa than in Europe.

#### Socio-Demographic Breakdown of Childcare and Support

We observed few statistical differences between academic parents' level of childcare compared to their socio-demographics, as can be seen from the Kruskal-Wallis significance test in Table 4. However, we noted a significant difference between the “ethnicity” of the respondent and “being worried about sending the kids back to school or daycare” (*p* < 0.009): the group of academic parents with Latino-Hispanic background showed significantly higher levels of worry to this question than the other parents (*post-hoc* analyses *p* < 0.05). We report a significant difference between the age of parents and the level of experienced childcare duties: parents between 25 and 45 have taken on more childcare duties than the parents older than 45 years old (*post-hoc* analyses *p* < 0.05).

**Table 4:** Kruskal Wallis significance test on differences between groups on childcare situation and support.

		**“I feel supported by my family”**		**“I am worried about sending kids back to school or daycare”**		**“I am satisfied with childcare situation” during lockdown**		**“I am satisfied with childcare situation” during reopening**		**“I have taken more childcare duties than before”**
	***H (df)***	***p***	***H (df)***	***p***	***H (df)***	***p***	***H (df)***	***p***	***H (df)***	***p***
Academic rank	4.259 *(8)*	0.833	14.087 *(8)*	0.080	15.089 *(8)*	0.057	12.504 *(8)*	0.130	7.774 *(8)*	0.456
Ethnicity	3.349 *(4)*	0.501	13.440 *(4)*	0.009*	7.245 *(4)*	0.123	4.169 *(4)*	0.384	4.062 *(4)*	0.398
Age	0.192 *(2)*	0.908	0.833 *(2)*	0.659	2.687 *(2)*	0.261	4.029 *(2)*	0.133	7.360 *(2)*	0.025*
Gender	2.849 *(2)*	0.241	3.516 *(2)*	0.172	0.408 *(2)*	0.815	3.493 *(2)*	0.174	1.386 *(2)*	0.500
Relationship	8.697 *(2)*	0.013*	1.058 *(2)*	0.589	1.783 *(2)*	0.410	0.499 *(2)*	0.779	0.335 *(2)*	0.846

*Bold values indicate results with p < 0.05*.

Apart from serving as a teacher, academic parents highlighted additional time challenges including: managing children's everyday activities, being a playmate or providing social interaction, arranging for physical exercises, and preparing lunch. These childcare demands vary depending on children's ages. For young children, parents need to be much more involved with the education process by providing technical or instructional support on virtual schooling or shifting to homeschooling altogether, which is challenging, as expressed by R282 (1 preschooler and 1 primary school-aged child): “*and for a small child virtual learning is very very hard*.” For older children, parents may need to provide support for the emotional isolation stemming from the lockdown, as expressed by R049 (1 primary school-aged child and 1 secondary school-aged child):

*The kids are home all day and they need people to interact with. They constantly interrupt my work. They are well intentioned and just want contact but it drains me. Further, the constant meal planning is a grind. I worry about their social integration in the future and one child had a falling out with their best friend near the start. She hasn't been able to repair that relationship and, as a result, has been extra lonely. We are trying to make this time happy for them while still managing our work and our own fears about job cuts. All our family is abroad so we worry about them also. My mother almost died in the shutdown due to depression and there was literally nothing I could do to help. AND, we have no one to help or relieve the pressure off us. The days have become monotonous and everything is very domestic. We are finding happiness with the little things and keeping our focus on our priorities and that, all in all, we are fine*.

#### Satisfaction Before COVID-19, During Lockdown, and at Reopening

**Figure 2:**
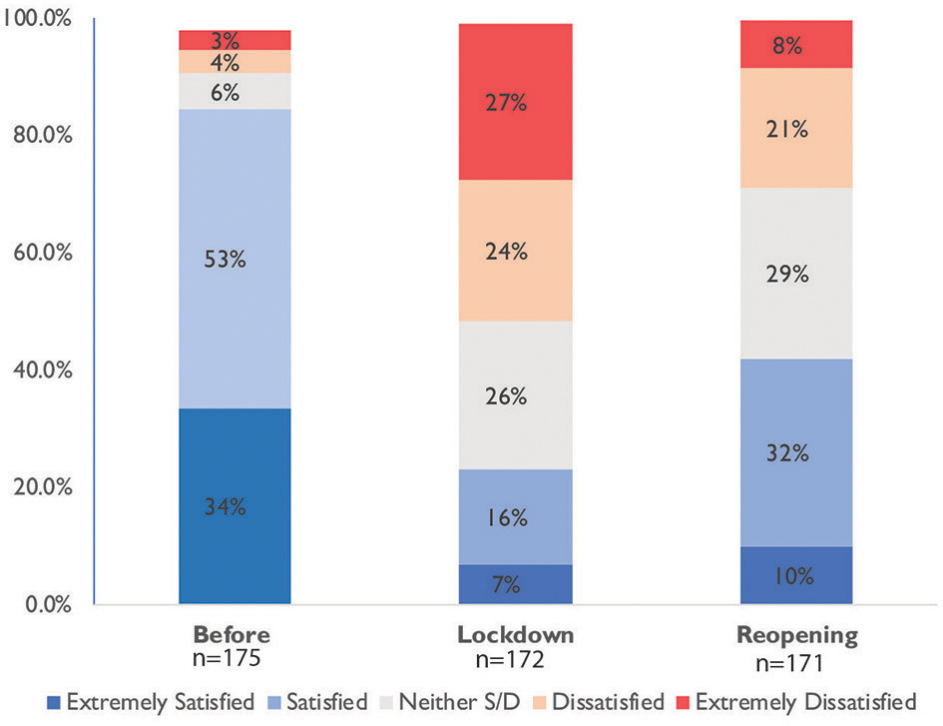
Childcare satisfaction pre- and post-lockdown.

We observe (Figure 2) that almost all academic parents were satisfied or extremely satisfied (89%) with their childcare arrangements before the pandemic, but more than half of all respondents (52%) became dissatisfied or extremely dissatisfied during lockdown. Childcare satisfaction during partial reopening is mixed: 29% dissatisfied or extremely dissatisfied, 29% neither dissatisfied nor satisfied, and 42% satisfied or extremely satisfied. Parents of younger children indicated a higher dissatisfaction rate about childcare during the lockdown: 44% parents of infants, 50% of toddlers, 59% of preschoolers, 52% of primary school-aged children, and only 32% parents of secondary school-aged children were dissatisfied or extremely dissatisfied.

**Figure 3:**
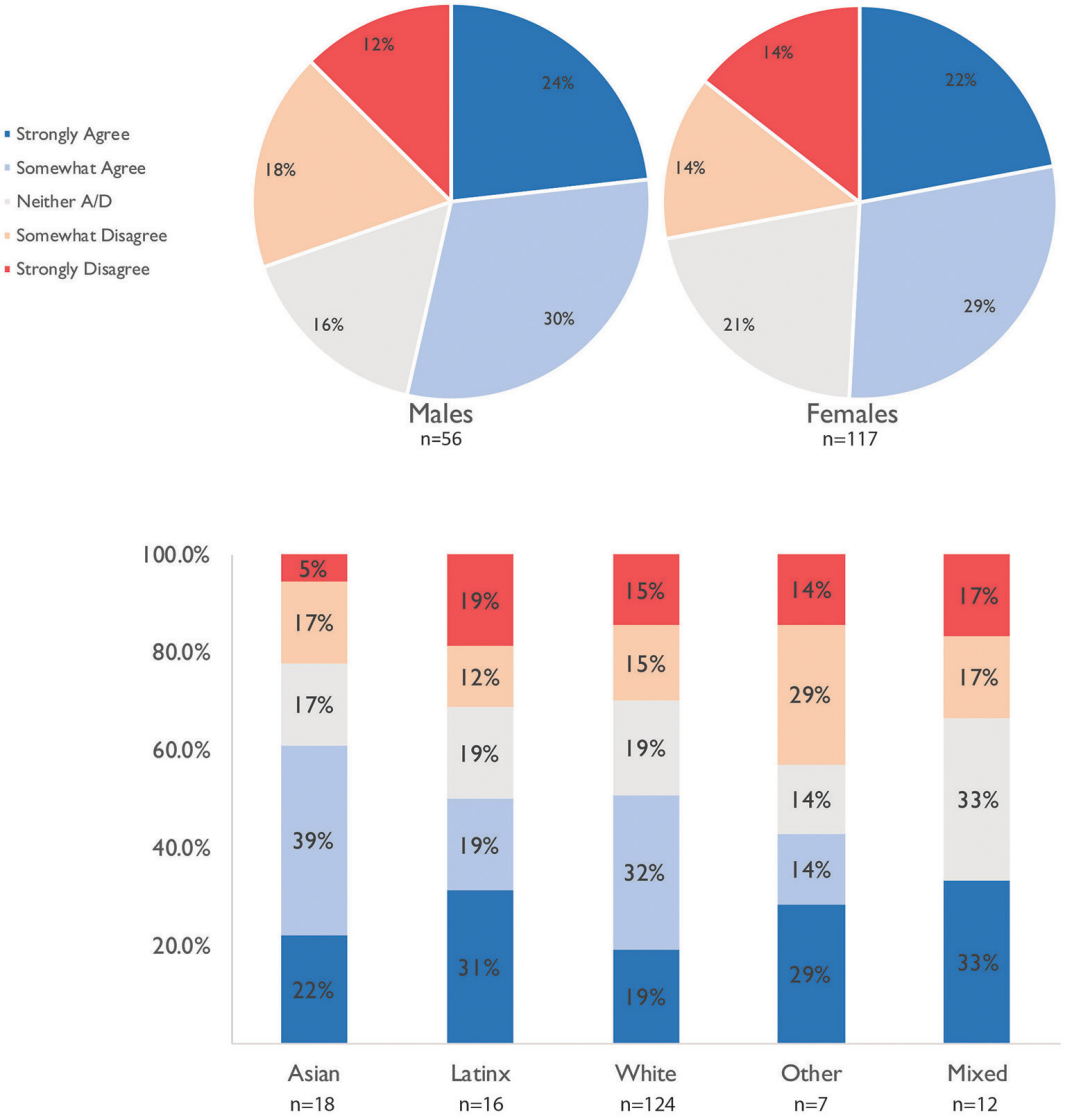
Academic agreement with the statement “I feel supported by my family as an academic parent” separated by gender and ethnicity, *n* = 177. *n* = 3 respondents self-identifying as “other” or “prefer not to say” gender not visualized.

Academic mothers and fathers reported feeling the same levels of support by their families (Figure 3), but there were differences in feeling supported based on ethnicity^4^, with Asian families feeling more supported. We also observe a significant difference in “relationship” categories (Table 4): parents living together experience more support than other parents (*post-hoc* analyses *p* < 0.05).

In some cases, grandparents have stepped up to help out, whereas other academic parents are not able to tap grandparents as a resource perhaps due to distance or for the risk of COVID-19 transmission, as expressed by R148 “*When things were loosened we could bubble up with grandparents next door but can't rely on them now that kids are in school and more exposed*.” Over half (58%) of respondents somewhat to strongly disagreed with the statement that they felt supported by their community. Academic parents, both fathers (80% of 56 respondents) and mothers (73% of 118 respondents), somewhat to strongly agreed that their co-parent had taken on more childcare duties than before. Table 3 also shows that the frequency of stay-at-home partners was dynamic and changed from 2% prior to the lockdown, to 18% during lockdown, and 9% during partial reopening. Academic parents have highlighted that they appreciated the added childcare their partner provided, as expressed by R054:

*As my husband and I have both been working from home our care load is now much more equitable (…). I have enjoyed this real sense of equality as before I did the majority of drop-offs and extra classes. I have enjoyed having less of a hectic schedule, less driving to different nurseries, we have saved money by not paying for nursery*.

#### Childcare Challenges

**Figure 4:**
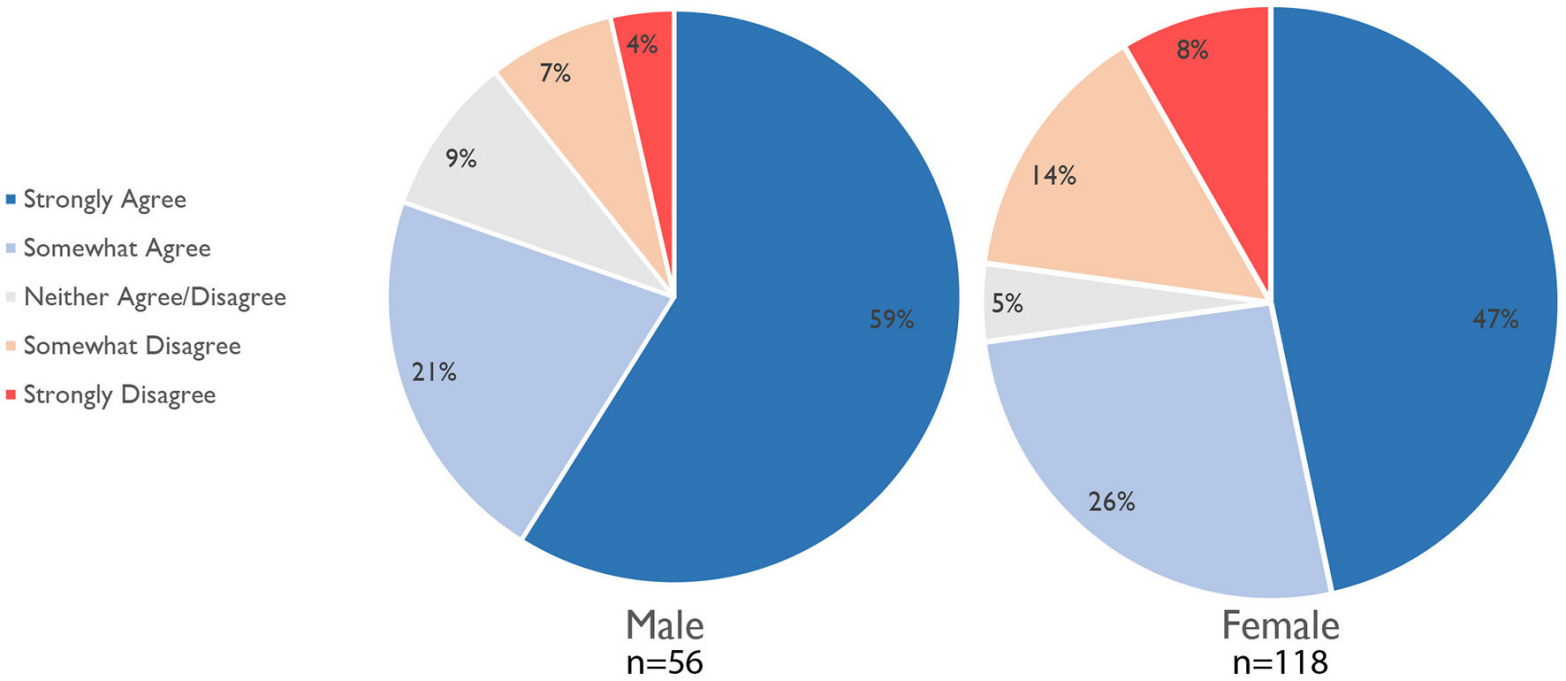
Agreement with statement “My co-parent has taken on more childcare duties during the pandemic than before,” for *n* = 177. *n* = 3 respondents identifying as “other” or “prefer not to say” gender not visualized.

Overall, academic parents agree that their co-parent took on more childcare responsibilities than before, with a stronger agreement for academic fathers than mothers, see Figure 4. Academic parents reported additional dissatisfaction when their co-parent worked outside the home, worked inflexible hours, or was the main breadwinner. Therefore, the academic parent had to serve as the primary and/or sole caregiver. For immigrant families, challenges arose when only the academic parent spoke the local language and by necessity had to take on more of the homeschooling and administrative tasks for the family.

#### Infrastructure Issues

Additional difficulties regarding access to infrastructure and restrictions to campus-based work was mentioned by about one-sixth of the participants as a key challenge. The impact of COVID lockdown on fieldwork and laboratory research was also mentioned as data collection had to be interrupted and, in some cases, caused significant research delays and setbacks. Extra challenges included access to infrastructure—books, campus, computer, etc.—and access to colleagues, research team and broader network. Respondents reported “*lab shutdown [and no] human subject research”* (R016) and “*loss of data”* (R215) as key challenges and highlight that “*being a parent means less time to deal with these things, less sleep, more stress”* (R016).

Disadvantages in using digital tools were highlighted and were mentioned by more than half of the respondents in the open-ended questions on use of digital tools and cloud environments. Internet dependency was an additional disadvantage associated with insufficient bandwidth as most members in the household needed internet access to perform their work and/or studies, as R073 (located in Ecuador) explained:

*We live in an area with many electric storms, which makes classes in the afternoon sometimes hard to accomplish. Internet issues are common when you have 4 people online at the same time all with zoom and graphics etc*.

Screen time and physical discomfort due to the long hours of computer work were raised as major concerns by a small number of participants. Respondents mentioned their workspace at home was sub-optimal and that, in summary, the whole online shift associated with parenting duties was overwhelming and tiring. Some pointed out that “all is bad.” A particular concern was how to guarantee quiet time for specific tasks that are not time flexible – e.g., scheduled committee meetings.

### Work Distribution and Well-Being

#### Challenges for Academic Parents During COVID-19

##### Work Distribution Due to Teaching From Home

Academic parents struggled with the impact of COVID-19 on their research, and to a lesser extent, on their teaching, see Table 5. Of the 113 respondents (completed surveys), over a third (37%) reported that the pandemic has extremely negatively impacted their research, with data collection and lab work identified as the most negatively impacted research tasks. For teaching, the percentage of respondents who indicated an extremely negative impact was less (9%). The fewer responses (*n* = 109) to this question may be attributed to fewer survey participants involved in teaching. The lower impact may be because teaching has been prioritized during the lockdown and therefore, has not been significantly impacted, as R034 indicated:

*My time in normal situations is consumed by five activities (…): research, education, outreach, being a parent / family member, social life. The outbreak of the pandemic and the lockdown meant that my very hands-on teaching had to move online, which took up all the time not being taken up by being a parent. So, during lockdown, there was only ‘being a parent' and ‘teaching (academically)’. My research was basically on pause as was my outreach activities and my social life. This is neither healthy (the social life) nor good for my career (the research part...)*

**Table 5:** Answers to the questions: “On a scale from 1-5 (very negatively – very positively), how has being an academic parent during the pandemic affected your research” and “On a scale from 1-5 (very negatively – very positively), how has being an academic parent during the pandemic affected your teaching,” showing percentages in different categories.

**Question**	***N***	**Extremely negative**	**Somewhat negative**	**Neither positive nor negative**	**Somewhat positive**	**Extremely positive**
Research in general	113	37%	40%	13%	9%	1%
Data collection	112	46%	30%	16%	7%	0%
Lab work	105^*^	50%	19%	29%	1%	1%
Analysis	113	38%	36%	18%	7%	1%
Reading	113	42%	29%	18%	9%	3%
Writing	113	42%	27%	16%	12%	4%
Dissemination activities	112	39%	28%	18%	13%	3%
Teaching in general	109	9%	38%	40%	11%	2%
Class preparation	109	16%	27%	44%	12%	2%
Lectures	108	13%	31%	45%	10%	1%
Asynchronous activities	107	13%	23%	46%	14%	4%
Grading	108	12%	21%	55%	9%	3%
Contact with students	108	26%	26%	33%	14%	1%
Supervision of students	108	19%	30%	38%	13%	1%

* *The lower n for lab work can be explained because not all respondents' research includes this component*.

Asynchronous activities were identified by 18% of respondents as somewhat positively or extremely positively impacted during the pandemic, while other teaching-related tasks were negatively impacted. The learning curve for teaching remotely and using new tools was considered an extra complication, as identified by R289:

*It's too much and overwhelming to be besieged by all these online teaching tools. In the end, all these teachers and lecturers will be compared in terms of their teaching prowess and out come teaching in competitions. Plus, you will be evaluated in your teaching by your students. Those who stick to boring ppt slides and old school teaching would probably get worse evaluation than those who are into teaching apps, making Youtube videos, powtoons, kahoots, edmodos, mindmaps etc. I feel suffocated by all these, and in the end feel so burnout just trying to prepare for a lesson. And not forgetting doing all these on the computer causes strain in my eyes and head*.

A major disadvantage of using digital platforms was an increase in workload or extended work hours as mentioned by one-tenth of the respondents in the open-ended questions. Workload was exacerbated by the repetition of tasks such as teaching online and face to face in some instances. The workload increase was attributed to the shift to online teaching as well as the lack of separation between work and home. As such, the longer work hours were not always imposed by the employee.

The negative impacts on research depended on the age of the child(ren): 33% of parents of infants, 39% of parents of toddlers, 34% of parents of preschoolers, 47% of parents of primary school-aged children, and 25% of parents of secondary school-aged children report that being an academic parent during the pandemic extremely negatively impacted their research. Similarly, the negative impact on teaching was greater for parents of younger children: 57% of parents of infants, 17% of parents of toddlers, 13% of parents of preschoolers, 2% of parents of primary school-aged children, and 0% of parents of secondary school-aged children reported that being an academic parent during the pandemic extremely negatively impacted their teaching.

We found a significant correlation between academic rank and the impact on teaching (*n* = 105): assistant and associate professors reported the greatest negative impact on teaching (58% of assistant and 76% of associate professors report a somewhat or extremely negative impact), as compared to Ph.D. candidates and researchers (18% of Ph.D. candidates and 0% of researchers report a somewhat or extremely negative impact). A possible explanation for this observation was that Ph.D. candidates and researchers had fewer teaching requirements than assistant and associate professors. Additionally, half (50%) of associate professors experienced an extremely negative impact on their contact with students, as compared to 21% of assistant professors and 25% of full professors. In terms of the impact on research broken down by academic rank (*n* = 104), the group that reported the greatest negative impact (52% extremely negatively impacted) were associate professors compared to 25% of assistant and 25% of full professors. Additionally, 17% of full professors reported an extremely positive impact on their writing productivity. A possible explanation for this positive impact was that the lab members and collaborators without children had more time to push articles forward, as expressed by R018:

*I'm benefitting from the time that childless students and collaborators have had to put toward publications. I am a co-author on several papers that are in the publication pipeline. Anything I am the lead on is very much on the back burner*.

##### Academic Performance Due to Work-Life Conflict

The impact of COVID-19 on experienced work productivity was directly related to academic rank (*n* = 112). The statement “it is harder for me to do my work duties as an academic parent than for my colleagues who are not parents” was found to be a function of academic rank: almost all (83%) Ph.D. candidates and post-docs (88%), and 76% of associate professors, vs. 58% of both assistant and full professors, strongly agreed to the same statement. There was a relationship between the statement “I have considered withdrawing from my program, resign from my program, or go part-time to be able to provide more childcare” and academic rank: half of all Ph.D. students (50%) strongly agreed vs. only 8% of full professors. Gender played a role as well when considering withdrawing from a program with 22% of the respondents self-identifying as men somewhat or strongly agreeing, vs. 40% of those identifying as women.

Providing additional childcare was a major challenge for academic parents during the COVID-19 pandemic. Almost all (82%) of the participants (*n* = 112) somewhat or strongly agreed that arranging childcare during the pandemic was stressful, and we found that almost one-third of the respondents to the open-ended questions identified managing work time and family responsibilities as a challenge for their research and almost half of them emphasized the challenge in balancing work and childcare. The magnitude of stress experienced was also directly related to the age of the child(ren), with over half the parents of young children (56% of parents of infants, 65% of parents of toddlers, 54% of parents of preschoolers, and 45% of parents of primary school-aged children) strongly agreeing with the statement that arranging childcare during a pandemic is stressful, vs. less than a quarter (21%) of the parents of secondary school aged children. This observation was also linked to the time availability of academic parents, with over half of all parents of young children (56% of parents of infants, 58% of parents of toddlers, and 63% of parents of preschoolers) and half (49%) of parents of primary school-aged children strongly agreeing with the statement “Being an academic parent has reduced my time availability for work duties during the pandemic,” vs. less than a quarter (21%) of the parents of secondary school-aged children.

The thematic analysis strongly highlighted concerns related to the long-term impacts of COVID-19 on academic work and performance, with the majority of respondents voicing their concerns. The main challenges related to managing work time and family responsibilities, meaning that these respondents had largely neglected research due to lack of time and prioritized teaching and supervision instead. Key concerns were, therefore, related to time management and lack of time for research, and issues with data collection. The key reasons for these were lack of quiet time and frequent interruptions.

The lockdown circumstances during the pandemic meant that “*it is impossible to write even a single sentence, either because [respondents are] exhausted or because someone calls for [them]”* (R056). As a consequence, “*analyzing and writing were almost impossible with constant interruptions from child, [and then] more errors, more work had to be redone. Fatigue, depression resulted”* (R048). The impact of parenting on the respondents' well-being during lockdown was related to exhaustion and fatigue due to the time demands of work and parenting combined which generates stress. Depression and mental health concerns were also mentioned by a minority of the respondents, although some pointed out that despite all pressures, they do prioritize their children and having the children around softens the situation and makes it all feel less stressful. Some academic parents also mentioned the difficulties associated with focusing on a video conference while a child walks in: *I can't really be in a meeting with colleagues while my child steps in asking me to make her a drink* (R017).

**Figure 5:**
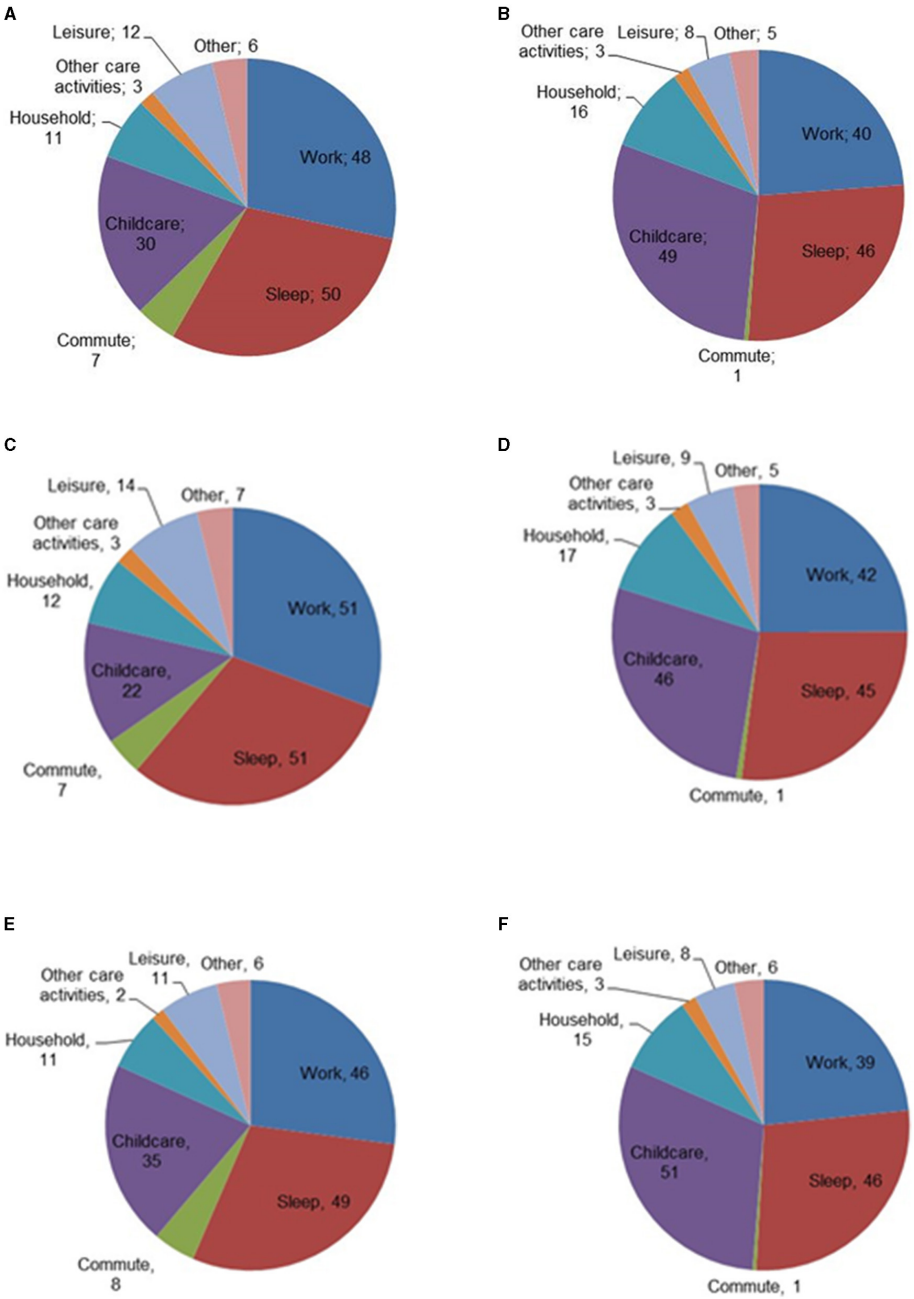
Average time (in hours per week) spent on various activities by academic parents: **(A)** before the pandemic; **(B)** during the pandemic, for completed surveys, *n* = 113. **(C)** before the pandemic, men; **(D)** during the pandemic, men (*n* = 41); **(E)** before the pandemic, women; **(F)** during the pandemic, women (*n* = 70).

To study how academic parents balanced their different tasks before and during the COVID-19 pandemic, participants were asked to estimate the amount of time they devoted on a weekly basis (168 h in total) in the categories work, sleep, commute, childcare, household, other care, leisure, and other. Figure 5 shows the time distribution on a weekly basis before and during the COVID-19 pandemic, based on 113 completed time estimates. Academic parents almost doubled the time they spent on childcare (76% increase) and household duties (74% increase) during the pandemic in comparison to before. Sleep was reported to decrease from an average of 7 h per night to 6.5 h per night, and 38 out of 113 respondents (34%) reported sleeping fewer than 6 h per night. Similar trends were reflected in the open-ended questions, for example R027 who notes that the least favorite part of the situation was the “*lack of sleep and self-care time*.”

In an additional analysis, we looked at the differences between those self-identifying as men (*n* = 41) and women (*n* = 70), see Figures 5C–F. From this breakdown, we note a similar decrease in time spent for men (18% decrease) and women (15% decrease). Overall, academic mothers worked 3 h per week fewer than academic fathers. On average, all academic parents slept less than before (6% decrease for academic mothers and 12% for academic fathers). Academic fathers on average slept more than academic mothers before the pandemic, however, both groups averaged comparable amounts of sleep during the pandemic (45 h for academic fathers and 46 h for academic mothers). Academic fathers reported spending more hours on household tasks than academic mothers before and during the pandemic. Academic mothers spent significantly more hours on childcare than academic fathers, both before and during the pandemic. Academic mothers increased the time spent on childcare during the pandemic as compared to before (46% increase), and academic fathers doubled the amount of time spent on childcare. Before and during the pandemic, academic mothers spent less time on leisure than academic fathers.

Work-life balance and time availability for academic work differed as a function of academic position, see Table 6. The average on a Likert-scale (1 = Strongly disagree, 5 = Strongly agree) for the statement “Being an academic parent has reduced my time availability for work duties during the pandemic” varied between 3.5 for lecturers and 4.8 for researchers. The teaching schedule of lecturers perhaps dictated this outcome. There was an effect of academic rank on the statement “Being an academic parent has reduced my publication output during the pandemic,” with an average value of 3.7 for the “other” category and 4.3 for Ph.D. candidates. Similarly, the statement “I have found a better work-life balance as an academic parent during the pandemic” had an average value of 1.8 for researchers vs. 3.2 for those with another academic appointment, and “My work hours are more flexible during the pandemic than before” with an average value of 3.3 for assistant and full professors, and 4.3 for researchers. The Kruskal-Wallis significance test, however, did not show a significance between the work-life balance questions and the academic rank of the respondent.

**Table 6:** Average outcome on Likert-scale (1 = Strongly disagree, 5 = Strongly agree) for work-life balance questions, broken down for academic rank.

**Question**	**Total**	**Ph.D. candidate**	**Researcher**	**Lecturer**	**Post-doctoral researcher**	**Assistant professor**	**Associate professor**	**Full professor**	**Other**
*N*	112	12	4	11	8	24	28	12	13
Being an academic parent has reduced my time availability for work duties during the pandemic	4.2	4.7	4.8	3.5	4.4	4.3	4.1	4.3	3.7
Being an academic parent has reduced my time availability for volunteering duties such as peer review during the pandemic	4.3	4.5	4.3	4.2	4.1	4.4	4.3	4.3	4.4
Being an academic parent has reduced my publication output during the pandemic	4.0	4.3	4.0	4.1	4.4	3.8	4.1	4.0	3.7
I have found a better work-life balance as an academic parent during the pandemic	2.8	2.4	1.8	2.9	2.9	3.0	2.6	2.8	3.2
My work hours are more flexible during the pandemic than before	3.7	3.8	4.3	4.0	4.1	3.3	3.6	3.3	4.1

*Other combines “other” and “other academic appointment”*.

There was a noticeable gender discrepancy between those experiencing additional satisfaction with work-life balance, with an average value of 2.5 on a 1-5 Likert scale for participants self-identifying as men (*n* = 40) and 3.0 for participants self-identifying as women (*n* = 70). This change from standard work hours to a more flexible schedule was similarly reflected in the open-ended questions, with academic parents reporting they now work non-standard hours, as expressed by R234:

*I need to fulfil all my duties related to teaching, organization and research career advancement like in ‘normal times’ while I have to provide care to my 2 little kids almost 24/7. I try to share the responsibilities, but they mostly fall on me. I work mostly at night / very early morning / sometimes weekends. I have problems often to cover childcare during my lectures*.

This flexibility goes hand in hand with a lack of separation between work and home so that “*work is 24/7 there is no separation”* (R244).

##### Home Office Ergonomics

Lack of adequate home office equipment and space presented a challenge impacting the overall well-being of academic parents. Respondents were concerned with the large time commitments associated with functioning as a parent-helper during their children's required online learning. Screen time for both parents and children was mentioned as problematic, as “*screen time restrictions are a thing of the past”* (R031) and participants reported to “*have far more headaches and eye strain, and [that their] kids do too”* (R056). The technology used for schools online teaching and learning was also pointed out as not ideal.

##### Overall Well-Being Due to Insecure Financial Status

In total, 96 survey respondents contributed their comments to the question about financial concerns. The job situation and market concerns were mentioned by more than half of them; slightly less than half of the respondents indicated they are already experiencing financial issues and one-tenth mentioned they fear for the future impacts of the current situation in their work life and ability to secure work. Moreover, some respondents indicated they were in fixed-term contracts, and that they feared for the lack or limitation of opening positions in the future. Instability and uncertainty in regard to the respondents' academic positions were the key and most mentioned theme, and a third (33%) of respondents shared this concern, including professors with tenure such as R056 (Associate professor) who stated: “*they will [be told to] come in 4 days a week, for 8 hours a day, like [they] used to, and if [their] children are not yet in school full-time, [they] won't be able to do that*.” In addition, contracts may not be extended and pay cuts due to the pandemic are a real concern. Reduced income was mentioned by one-sixth of the respondents and a minority said their spouses lost their job or other sources of income were reduced, as reflected by R052: “*family business has suffered a significant loss [and they] also highly suspect [their] academic position will be made redundant sometime soon.”* Being a single income family adds extra pressure and some were already facing financial issues, while immigrants mentioned the added concern about losing their jobs and having to leave the country where they currently live.

Due to reduced institutional funding, some respondents raised concern about the need to self-fund their research and travel so they can have a competitive CV to apply for future academic positions. However, due to the need to perform extra work to compensate for reduced income or spouse's loss of work, respondents indicated they have embraced extra roles and therefore have reduced time to work and look after their children.

Fixed-term contracts and an increasingly difficult job market added financial stress, as R043 (Ph.D. candidate) explained: *[There are] even fewer job perspectives than before because budgets and positions have been eliminated. Not having enough time to write and publish will affect my career and thus my financial situation*. Other financial pressures mentioned included being “*100% grant funded, so the inability to secure additional grants/stay productive means [they] are out the door”* (R185), and also the “*worry about [the current situation having an] impact long term if grant review panels punish those who are parents because of the lost productivity”* (R244).

One-third of the respondents reported they were unaffected, most likely as they were in a tenured position and did not feel threatened by the job market instability.

#### Opportunities for Academic Parents During COVID-19

The negative impact of the COVID-19 lockdown on work distribution and work-life balance was significant; however, there were opportunities identified by the respondents of our survey. Mentions of “opportunities” in the thematic analysis were significantly less frequent than the “challenges,” and almost half of the respondents did not see any advantage in the lockdown situation. However, some new opportunities were highlighted. For instance, respondents repeatedly mentioned increases in productivity, access to resources and new opportunities, and improvements in personal life and well-being. Survey respondents identified the time saving of working from home as an opportunity. New research opportunities (and funding opportunities for this type of research), ideas and collaborations – particularly in COVID-related research were enabled by the lack of geographical boundaries in the context of largely working online. More reading and writing time were identified, albeit at times limited by noise and lack of quiet time. Publications were produced as the reduction of meetings and commute time enabled researchers to re-focus. Additional mentions included more time for students, extra time for unusual tasks and, interestingly, the opportunity of capitalizing working time on childless students and collaborators who have more time (see also section Work Distribution Due to Teaching From Home).

##### Increased Work Productivity Due to Less Traveling

In total, 108 survey respondents commented on the question regarding the impact of COVID-19 on traveling. The time saved in commuting was frequently mentioned in our thematic analysis as by not commuting, respondents highlighted they had more time to dedicate to family, they saved money, and they experienced improved well-being and less stress as they managed to add more exercise into their routines. Additional time spent at home meant parents could be more present and involved in the education and daily lives of their child(ren), as R199 explained: “*Time usually spent commuting is now put into cooking healthy dinners and doing chores. My child knows I am at home all the time, so it is good to be 'present' for him.”* This closeness to family helped in finding an overall sense of purpose and direction, despite the hardship of the pandemic and lockdown circumstances. The opportunity to “*do the best out of it and enjoy the time together (…) growing closer as a family (…) [allowed for] personal growth in taking life choices and responsibility*” (R220).

Online conferences were viewed favorably by survey respondents due to accessibility and the money saved both in travel and commute. Not traveling was also viewed as a positive as it helped to manage family life, and it was reported to help reduce and help manage stress. The aspect of helping to manage family life is reflected by R062:

*Pre-pandemic, my parental responsibilities limited my ability to travel to conferences, to serve on panels, etc. My partner is an on-call physician, which makes it very difficult for me to be gone - even for day trips. I have been able to attend many virtual conferences [during the pandemic]*.

Online meetings were seen as an inclusive opportunity to allow people from regional and remote areas to participate in meetings and conferences. Finally, respondents valued the sustainability aspect of traveling less and the flexibility of working from home and attending conferences at the same time. The ability to attend and deliver seminars and conferences remotely was also identified as an opportunity for Early Career Researchers who did not have sufficient funds to attend international conferences. As such, online platforms enabled their participation and extended their academic network. Overall, the ability to attend conferences virtually was an opportunity due to the sustainability advantages and lower costs, as explained by R215 (located in India):

*(…) Being able to attend multiple international conferences, while I could barely afford 1 in a normal year. Being able to be on multiple panels and give many webinar talks that were well attended. My child has also been able to access many online reading and cultural fests, which was a good experience*.

It is also worth noting, however, that not traveling was also mentioned as a challenge by over half of the respondents. These responses highlight that, even though online meetings and conferences save commute and travel time, they were also viewed as impersonal and therefore not ideal. An additional challenge was presented when allocating work and planning for multiple schedules in multiple time zones, as these can be incompatible with dedicated family time. Some respondents said they missed their extended family, the quiet and personal moments of traveling for work, and travel in general. In addition, respondents reported missing the stimulation, networking, and new ideas that emerge both from face-to-face conferences and daily encounters with colleagues. In this regard, not attending conferences was highlighted as a disadvantage, for example, as they “*no longer travel which is advantageous in maintaining home responsibilities but detrimental for developing collaboration”* (R043). In addition, as R030 explains: “*Not having to travel is great. Not getting to travel is awful. I miss the peace & freedom of a hotel room to myself. It was important time where I felt I could recharge.”*

##### Use of Digital Tools

In total 98 respondents commented on the question about the use of digital tools and cloud environments, and themes related to opportunities in regard to the use of digital tools were mentioned by three quarters of them. These opportunities relate both to work and to the relationship between parents and children.

In regard to work, the main advantages identified were accessibility – online meetings and access to resources and colleagues, learning new tools and reaching a significant level of technology proficiency, flexibility in work hours and location, and the possibility of working from home. Some respondents felt they could more easily and quickly communicate with students through online means rather than waiting for meetings, and some mentioned they felt more productive as they can multitask in zoom meetings. Asynchronous teaching was seen as an advantage under the lockdown situation, as academics could choose the best time to record their lectures, and then could do so when the children were sleeping. Indeed, the flexibility of online and, in particular, asynchronous teaching was essential for working around parenting responsibilities and “*has allowed [academic parents] to continue teaching since [they are] able to do most of it at night when the kids are in bed”* (R018).

Other advantages mentioned were shorter meetings, as the meetings contained only what was required rather than extra conversations before and after, sustainability due to reduced travel, improvement in teaching quality due to more time for preparation and more time to students, as well as work quality in general for similar reasons. Further opportunities identified during the COVID lockdown period related to learning new digital tools and skills, moving toward paperless workflows (*It forces the institution to go for paperless earlier and this is what I have advocated for long* – R265), and having time to undertake unusual tasks, as R189 highlights: “*I find myself doing lots of things like this survey or being on panels.”*

Children walking into videoconferences was framed positively by some academic parents “*My kids entering the room happy during zoom meeting is certainly a happy memory*” (R009), and was seen by some as an opportunity to get to know their colleagues better as it favors “*a more personal relation to work colleagues - being able to bond with other academics and collaborators over our parenting-work-struggles*” (R158).

The use of screen time to be able to work was framed positively by some academic parents as an ability to assist homeschooling and the child's language development, as expressed by R283 (located in Colombia):

*Turn on the TV in English to let your kid be bilingual. His English has improved a lot. Now, he can communicate with full sentences in English thanks to the amount of exposure to the language. Do not feel guilty for letting them watch TV. There is a pandemic and all of us are trying to survive*.

##### More Flexibility and Work Life Balance

Opportunities for flexibility and work-life balance identified in thematic analysis regard re-focusing priorities and better connecting with their families, finding time for self-development and personal growth, bonding and empathizing with colleagues, particularly with other colleagues who are also parents. Some respondents also mentioned they found an increase in their self-confidence due to the challenges of navigating the current situation while being a parent and that they found rewards from sharing their skills with others who benefit from them – e.g., online work.

**Figure 6:**
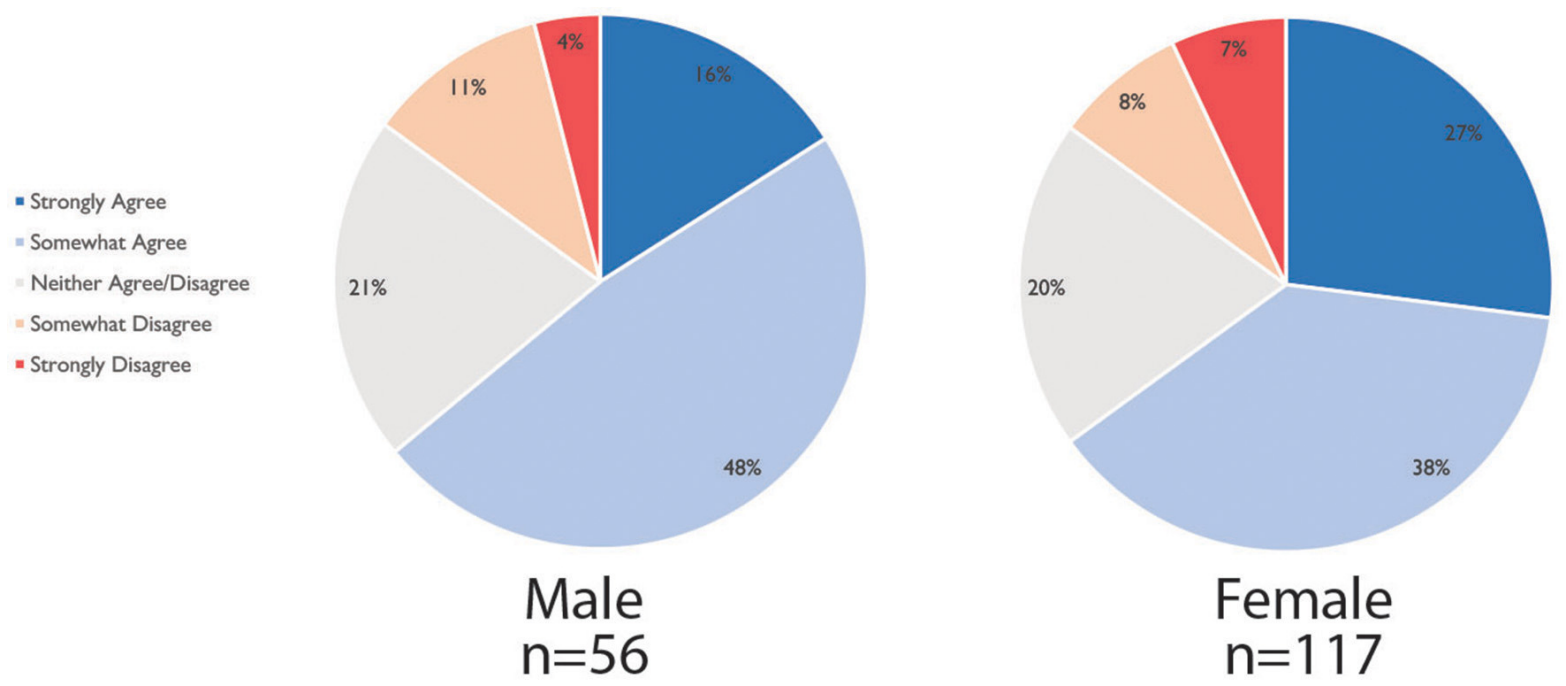
Agreement to statement “I feel more involved in the education of my children as a result of the pandemic,” by gender, partially completed responses, *n* = 176. *n* = 3 respondents self-identifying as “other” gender not visualized.

The pandemic provided academic parents additional opportunities to be directly involved in the education of their children (Figure 6). On a 1-5 Likert scale, an average value to the statement “I feel more involved in the education of my children as a result of the pandemic” was 3.7, for the partially completed responses, and a small difference in gender, with over a quarter (27%) of those self-identifying as women strongly agreeing with the statement as compared to 16% of men. There was also an effect of ethnicity, with less than two thirds (61%) of white academic parents agreeing or strongly agreeing to this statement, as compared to three quarters of Asian parents (79%) and of Latinx/Hispanic parents (73%). Respondents also saw opportunities for teaching and learning from their own children (3). Participants mentioned the benefits for children better seeing and understanding what their parents do for work, and even participate in it, as R182 explained: “*[My] daughter got involved in one of my pre-recorded virtual conference presentations*.” In addition, they “*can share all the learned skills with [their] children, [and] sometimes [be] helped by them”* (R077). An added advantage was that school meetings and discussions with children's teachers were better facilitated now that it was easier to fit it into the online work schedule.

The ability to see the successes of their children was considered an advantage as well “*It made me happy that I got compliments from the teacher responsible for his development that he advanced so much during this period”* (R081). Academic parents had the chance to better understand the learning difficulties their child(ren) experience and get “*better insight in the[ir] educational needs*” (R019). Other academic parents mentioned that they appreciated the ability to teach and “*give children some responsibility*” (R216), and to be more independent.

Academics whose partners had flexible work schedules and were working from home enjoyed the flexibility offered by the situation, as expressed by R053:

*The distance learning was flexible, they got a schedule for a whole week but they were not obliged to follow it day by day. So, we often crammed all tasks in just a few days. I usually worked in the morning, then we did school work in the afternoon and I worked a sort of evening shift while my partner did household stuff (…). On days when we didn't do school work I did somewhat more normal hours. Or we didn't and went on a hiking trip in the afternoon if we felt like it. Both of our jobs are flexible like that*.

This new flexibility allowed academic parents to find solutions to face the challenges discussed in sections Work Distribution Due to Teaching From Home and Academic Performance Due to Work-Life Conflict. The categories for solutions academic parents have used are: (1) psychological adjustment, (2) developing schedules, and (3) setting up a functional work space. In the category of psychological adjustment, we identified solutions related to accepting the situation, not being to hard on oneself, lowered expectations for parenting, lowered expectations for academic work (R003: “*Limit expectations and strive to achieve basic minimum standards. Acknowledging the difficulties in the situation*.”), and practicing patience. In terms of developing schedules, participants mentioned organization, planning in advance, taking turns with co-parenting, developing routines, dividing work into smaller, more manageable chunks of time, and setting boundaries with the children. Some academic parents reported that rigid scheduling did not work for them, while others acknowledged the unpredictable nature of the situation: “*Making and sticking to routine and prepared to make spontaneous compromises*” (R007). Finally, the need for a private space while having children at home shifted from a challenge (“*I have taken meetings from the back seat in my car, parked on the street, sometimes*.” – R030) to an opportunity to improve the home office, including mentions of being able to switch to a standing desk.

## Discussion

### Gender Differences

Gender influenced just three categories within this study (impacts to work-life balance, feeling involved in the education of the children, and considering withdrawing from the program or going part-time), as well as the reported time spent on work in the time logs. Beyond these categories, we did not observe differences between the experiences of academic mothers and fathers. Our findings shed new light on the focus on academic mothers found in the literature and call into question the validity of solely focusing on the experience of academic mothers, although it is important to note that direct comparisons cannot be made. Previous research (Comer and Stites-Doe, 2006; Isgro and Castañeda, 2015; Moreau and Kerner, 2015; Trussell, 2015; Bomert and Leinfellner, 2017; Hertlein et al., 2018; Huppatz et al., 2019) focused on how motherhood influences academic careers, as well as how women have published less and submitted fewer proposals during the pandemic (Squazzoni et al., 2020; Viglione, 2020). On the other hand, van Engen et al. (2019) found that both academic mothers and fathers adopt similar strategies to negotiate their professional identity within the discourse of hegemonic masculinity, which affects both academic mothers and fathers, provided that they are actively involved in caregiving.

Our research focused on all academic parents and we found that the challenges faced by combining academic work and childcare during the COVID-19 pandemic are similar for all academic parents regardless of their gender. In fact, some academic fathers with full-time working partners have indicated that colleagues with stay-at-home partners do not understand their current struggles, as reflected by R062:

*My department has paid lip-service to my situation by saying things like “enjoy this time with your kids” while simultaneously providing criticism on my tenure package and production. I have not been penalized or reviewed poorly by anyone in the administration, but my experience is a near constant complete lack of empathy or understanding for my situation, particularly as a male acting as the primary caregiver. The men in my department do not understand at all, I suppose, because their lived experience was so different. My department is predominately men*.

The findings of our time logs are in line with those by Deryugina et al. (2021): all academic parents report a loss of time spent working, and academic mothers have less available time for working during the pandemic than academic fathers: 3 h per week of difference between academic mothers and fathers during the pandemic in our time survey, vs. 30 min per day of difference in research time between academic mother and fathers in the time survey by Deryugina et al. (2021). Our survey studied time on a weekly basis, whereas (Deryugina et al., 2021) studied time on a daily basis, and our study looked at “work” as one time category, whereas (Deryugina et al., 2021) looked at research time. Regardless of these differences in methods, the outcome is comparable.

The finding that gender did not play a critical role is perhaps surprising and future research could explore how care-taking roles of academic parents currently influence their careers, how these care-taking roles may have shifted over the past decades, and study a larger number of academics to see if our findings were a product of self-selection of the study participants.

### Influence of Academic Rank

A number of the studies analyzed in the literature review focused on specific academic ranks: students (Moreau and Kerner, 2015), doctoral students (Springer et al., 2009; Mirick and Wladkowski, 2018; Wladkowski and Mirick, 2019), the tenure-track years (Comer and Stites-Doe, 2006; Poronsky et al., 2012; Trussell, 2015), or mid-career academics (senior lecturers and associate professors as studied by Harris et al., 2019). Other studies (Rafnsdóttir and Heijstra, 2013; McCutcheon and Morrison, 2018; Huppatz et al., 2019; Moreau and Robertson, 2019; van Engen et al., 2019) included academics from different ranks, but did not consider this variable in the data analysis.

The findings from our study differed from other studies regarding academic rank and the impact of COVID-19 (e.g., Squazzoni et al., 2020, a bibliometric analysis of authorship and author order by gender of articles submitted during the first months of the pandemic) in a few key ways, which may be explained by the differences in study approach, specifically: (1) the last author is not always the more senior member, as in some cases authors decide to use alphabetical order, or the last author may be from the funding body, (2) the study by Squazzoni et al. (2020) only looked at gender, not at the influence of being a parent, and (3) our study looked at research, teaching, and work-life balance, and did not focus on publication output.

Our study did not find that the impact of COVID-19 on the work of academic parents changed as a function of seniority (note that there was not a direct relation between academic rank and the age of the respondents' children). We observed that assistant and associate professors experienced the largest negative impact on their teaching. Associate professors also experienced the largest negative impact on their research. The groups who found it most difficult to fulfil their work duties during the pandemic were the Ph.D. candidates, post-doctoral researchers, and associate professors. We can only speculate why these groups were most impacted, but possible reasons are: (1) Ph.D. candidates and post-doctoral researchers experienced delays in their research as a result of the pandemic, especially related to lab or field work, and (2) associate professors may be struggling with the broader portfolio of responsibilities and larger administrative and service loads than assistant professors. This observation is in line with the finding by Gewin (2021), who reported that mid-career academics experienced the highest level of stress during the pandemic.

Future research is necessary to further explore the particular difficulties the different groups face, and how these have impacted their academic work, as well as to explore the particular challenges associate professors face.

### Recommendations Moving Forward in The New Normal

The lessons learned from this research, and from this difficult period, offer a unique opportunity to rethink current practices in the university setting as we move toward a new normal, as expressed by R158:

*Relax into it and radically, consciously, let go of or challenge impossible expectations – my own, those of my field, my workplace/employer, and society as a whole. I found that rising very early – not something I did regularly before the pandemic – helped me immensely in preserving my sanity, having some alone time even during strict lockdown, learning new things, nourishing a hobby and at times get work done – but the latter should not be the main focus of those early morning hours, I've learned. My experience of the pandemic has also shifted as time has passed – at first it was an adventure for the kids, then it was a chance to spend more time near relatives we rarely see, then it grew tiresome, then frustrating, and right now I feel that we have found peace with our current situation. Another thing that's really helped me is the recognition (and, dare I say it, relief!) that we are in a time of transition away from an unhealthy, untenable system of work and family life, toward something as yet undefined. This is the time to make the first steps toward the changes that are so urgently needed so that parents can find ways of not just wrangling work and childcare and family life, but be supported by their employers, communities and society. As such, I am taking this time to consciously think about the arrangements, support systems, and educational model I want for my children and family, and I hope many other parents are taking this time to dare to think outside the box as well – because the future is here for us to shape it, not to stumble into it with a COVID hangover, prone to being rushed and pushed into a convenience mold thought up by politicians or others who are removed from the realities of (academic) working parents/families*.

The pandemic impacted academics of different ranks in a variety of ways and as such, we recommend employing strategies for academic parents tailored to the stage of their careers. For example, early career researchers are still on the learning curve for balancing work and life, conducting research, writing for publication, developing their courses, and at the same time, have less financial security and often no permanent contracts. Full professors may be more experienced in managing their time, and possibly can delegate tasks to their team, have more teaching material developed over the past years, as well as have more job security, but may be dealing with more complex administrative and service questions. As such, a single policy for support might indeed benefit one stage (e.g., an early career scientist) while providing little to no support (or even causing additional hardships) for faculty in other career stages.

A certain level of flexibility is required (O'Brien Katharine et al., 2015) instead of implementing a “one size fits all” approach (Windsor and Crawford, 2020). This need for “tailor-made” solutions aligns with our finding that the needs of academic parents widely differ and that academic parents of different ranks, from different fields, and with different ages of children benefit from varied types of support. Indeed, tailor-made solutions can mean adding time to the tenure clock for some academics, as well as offering young faculty the opportunity to go up for promotion early if the opportunity arises (Gibson et al., 2020).

It's time for universities to turn the advice from their emails into implemented policies. It is important to give academic parents a voice in the administrative process of developing policies regarding impacts of COVID-19 on academic parents, possibly through a “…/… *working parent task force …/…*” (R011). The new normal presents an opportunity to create more individualized profiles for academics with individualized tenure and promotion requirements. The shift to remote teaching has demanded a large time investment for academic parents who teach and the subsequent change toward combined on-campus and online teaching poses additional challenges; therefore, the help of extra teaching assistants and/or additional support for teaching becomes necessary (Andersen et al., 2020) with a priority to support those who have the highest caretaking loads (Kramer, 2020). A temporary future release of administrative and teaching loads to refocus on research could help those academic parents whose research has stalled completely during the pandemic.

Changes in support systems as provided by our universities could mean that universities embrace a “culture of care,” as identified in the literature review (Springer et al., 2009; Moreau and Kerner, 2015; Mirick and Wladkowski, 2018; Wladkowski and Mirick, 2019). Examples of measures within a culture of care are (Comer and Stites-Doe, 2006; Ward and Wolf-Wendel, 2012; O'Brien Katharine et al., 2015): having an open conversation with faculty about their preferred hours of teaching, providing work-from-home accommodations when applicable, letting faculty teach topics they are passionate about and hold service appointments that align with their skills and purpose, offering extended leave and release time options, more flexible tenure clocks and adjusted requirements for making tenure, on-site childcare, an appointed staff person to oversee work-life balance issues for the campus community, and having clear and transparent communication about policies and procedures. Where university administrators implement family-friendly policies, compassionate-related policies (Deliens and Van Den Bossche, 2019), or focus on the individual rather than publication metrics (Redden, 2019), the work and personal lives of academic parents (Kossek and Ruderman, 2012) and all individuals (Isgro and Castañeda, 2015) on campus are improved, and these universities become attractive employers for recruiting top talent (Schiebinger et al., 2008; Harris et al., 2019; Huppatz et al., 2019).

This shift requires administrators to let go of ultra-competitive, neoliberal practices that lead to the loss of talent (Gibson et al., 2020), and turn universities into a place where we prepare young people to solve the challenges of the 21st century and to invest in human capital in an inclusive way (van Engen et al., 2019). Additional training may be necessary to teach administrators how to think in a more inclusive way, as was recommended for student-parents in the past (Springer et al., 2009). Awareness is key here and administrators need to understand the difficulties academic parents face to be able to enact changes (van Engen et al., 2019). It's also important for administrators (Perlmutter, 2020) to show care in tone and manner, which is even more important in stressful times such as a pandemic, and to learn to look for an alternative path to grant the requests of staff. Being open and vulnerable to their staff is important for administrators as well, as showing vulnerability about the challenges administrators have faced as academic parents themselves changes the culture (Reisz, 2020).

The COVID-19 pandemic has taught us that remote work and remote meetings are possible and we anticipate that a certain number of meetings/conferences will remain virtual, as more academics question the carbon footprint of travel (Spinellis and Louridas, 2013). As this shift increases the amount of time academic parents spend behind their computers, a way to rethink conferences can be to include wellness components (such as online Zumba or yoga classes to encourage attendees to get up from the computer and get active) and deliberate fun social components (a shared game to replace a networking event).

In addition to these recommendations for policy and broader reflections on the new normal, we found that academic parents need time and space to meet their individual needs: besides being an academic and a parent, they need to be able to develop a sense of individual self – a need that was sometimes met in the past by traveling alone to a conference. This need is even more pressing now, as the boundaries between work and home have blurred, and academic parents are sometimes juggling both roles concomitantly. The COVID-19 pandemic is expected to have a lasting impact on conference and work-related travel, and we need to rethink how academic parents foster their identity of self in the midst of their work and parenting demands.

## Summary and Conclusions

We used literature review and survey data with quantitative and qualitative analyses to answer “How has the COVID-19 pandemic affected the research and teaching of academic parents?” and “Which opportunities and challenges has the pandemic presented to academic parents?” From the literature review, we learned that academic mothers face a motherhood penalty in their career, which is not experienced by academic fathers. We also learned that women academics' publication output dropped during the initial lockdown stage of the pandemic and that higher educated women in the labor force spent the largest number of hours on homeschooling their children.

We designed a survey around the themes that emerged from the literature review to study the impact of the COVID-19 pandemic on academic parents including: childcare before and during the pandemic, work as an academic parent during the pandemic, overall time availability and work-life balance, and university support for academic parents. The survey contained 41 questions in a combination of multiple-choice questions, Likert-scale questions, and open-ended questions. The analysis methods we used were quantitative analysis (statistical analysis, cross-tabulation, and statistical tests) and qualitative analysis (inductive thematic analysis), which allowed us to identify the main challenges and opportunities for academic parents during the COVID-19 pandemic, and identify which socio-demographic aspects most influenced the outcomes.

**Figure 7:**
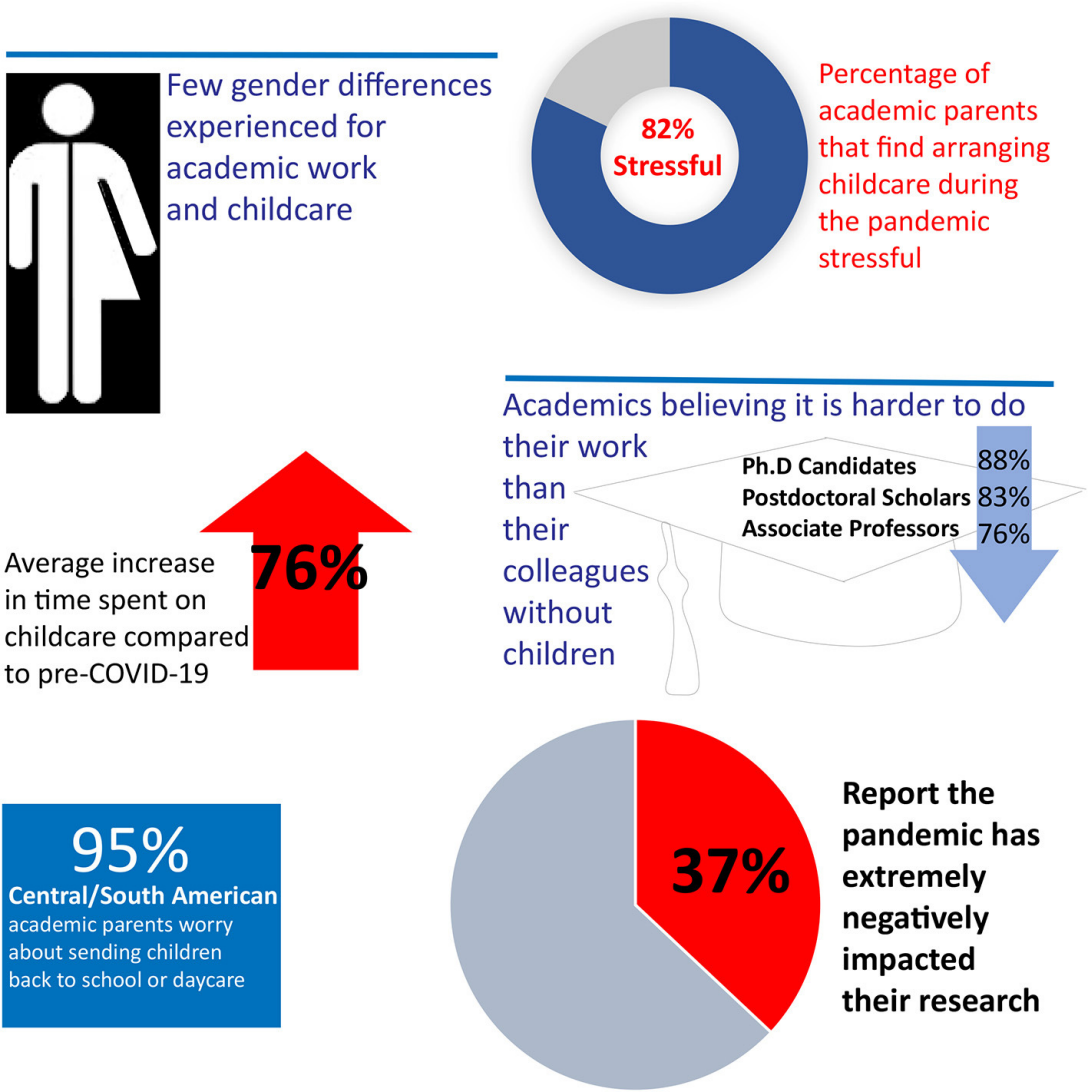
Graphical summary of main findings.

The main challenges we identified for academic parents during the COVID-19 pandemic were balancing the combination of academic work and childcare when regular childcare options were largely reduced. Additional parental challenges included managing the burden of providing or supporting children's education while also catering to the physical and social well-being of children in lockdown. Key work challenges included reduced availability for doing research as preparing for remote or hybrid teaching takes more time, managing online meetings and teaching with children around at home, lack of focused time for writing, uncertainty with regard to fixed-term contracts and funding availability, negative impact on personal well-being, and challenges related to the lack of infrastructure access and inadequate workspaces in the home environment (Figure 7). Identified opportunities included: new research on the topics related to the pandemic, opportunities for international collaboration, the ability to attend conferences virtually, becoming more involved in the education of the child(ren), increased time for reflection and finding an overall sense of purpose and direction as a result of these tumultuous times, and saving time that is normally spent commuting. The conclusion of the survey is that the pandemic has had an overall negative effect on the research of academic parents, and to a lesser extent on their teaching and overall work-life balance. University policy changes in support of academic parents have lagged behind promises and advice presented in emails.

In analyzing the open-ended questions, we learned that the same “challenge” may be interpreted differently by individual respondents. The childcare “challenge,” for example, was seen by many academic parents as a source of stress, but framed by others as an opportunity to bond with their children. These varying interpretations demonstrate that lived experiences and personal perspectives matter, underlining that a “one size fits all” solution for academic parents is not recommended. We speculate that the different reactions may be a combination of personal aspects such as mindset, the individual's response to change, and workplace and national culture.

We focused on the impact of the COVID-19 pandemic on all academic parents and found, on average, similar results when breaking down the survey responses by gender, although we also found that academic mothers reported working fewer hours during the pandemic than academic fathers. We found, however, differences in outcomes by academic rank, with associate professors experiencing the largest negative impact on their academic work. Academic parents of secondary school-aged children struggled less with childcare and academic work during the pandemic than those of younger children, but were still negatively affected. We recommend that university support should consist of tailor-made or elective measures catering for each individual's needs. Our recommendation is to provide space for compassion on different societal roles (academic, parent, informal caregiver, bereaved…), reflected by university policy changes. As we move toward a new normal following the COVID-19 pandemic, adaptability is essential: academics and universities can take the lead in developing systems (for childcare, work or home environment) and applying new policies that cater to personal needs, and that create a more compassionate workplace and society. This approach will improve not only well-being and productivity in the recovery months after the initial lockdown, but will also prepare for any future national or global emergency.

While this study received considerable international participation, we did not receive any completed responses from academic parents identifying as Black or Indigenous/First Nations, and from academic parents from the African continent nor from Southern or Eastern Europe. We noticed a difference in participation of Asian and Latinx parents between partially completed and fully completed surveys. Similarly, the responses from single parents and parents who have more fluid gender identities were very low. We acknowledge that the length of the survey may have selected for parents with fewer time constraints. Similarly, we found a higher percentage of survey discontinuation from academic parents who were dissatisfied with their childcare arrangements during lockdown and partial reopening. We did not receive enough responses to study the effect of the different national policies during the pandemic, and the adaptation of universities to these measures. We did observe that concern levels about sending children back to school differed by region, which could be related to different measures as well as access to and local quality of healthcare. We recruited participants through our own social networks and by email, and there may have been a self-selecting bias among the participants. Future research should take care to increase participation of the underrepresented groups.

## Data Availability Statement

The datasets presented in this study can be found in online repositories. The names of the repository/repositories and accession number(s) can be found below: https://zenodo.org/record/4384743#.X-IFQ6Z7lEY&lt.

## Ethics Statement

The studies involving human participants were reviewed and approved by IRB approval nr 2020-056M obtained through Universidad San Francisco de Quito. The patients/participants provided their written informed consent to participate in this study.

## Author Contributions

EL: conceptualization, literature review, IRB application, survey design, quantitative analysis, qualitative analysis, manuscript writing, and project management. YT: qualitative analysis, project management, and manuscript writing. ST: literature review, qualitative analysis, and manuscript writing. KL and EP-T: quantitative analysis and manuscript writing. All authors contributed to the article and approved the submitted version.

## Conflict of Interest

The authors declare that the research was conducted in the absence of any commercial or financial relationships that could be construed as a potential conflict of interest.

## Publisher's Note

All claims expressed in this article are solely those of the authors and do not necessarily represent those of their affiliated organizations, or those of the publisher, the editors and the reviewers. Any product that may be evaluated in this article, or claim that may be made by its manufacturer, is not guaranteed or endorsed by the publisher.

## Figures and Tables

**Annex 1:** The table below shows the codes and themes derived from the question “Which challenges have you encountered in your research during the COVID-19 pandemic? How did being a parent relate to these challenges?”

**Theme**	**Sub-theme**	**Mentions**
Academic work and performance	Managing work time and family responsibilities	29
	Neglect/no time for research	23
	No quiet/reading and writing time	23
	Data collection	22
	Interruptions/distraction	15
	Parenting time consuming/exacerbates challenges	8
	Prioritize teaching/supervision	7
	Increase in workload	5
	Low productivity/progress	3
	Shift to online	3
	Noise	2
	Motivation	1
	Feeling organized	1
	Online supervisions	1
Total		143
Personal/parent well-being	Exhaustion/fatigue	5
	Stress	5
	Finances	5
	Depression/mental health	3
	Uncertainty re childcare	2
	Seeing students challenges as a parent	2
	Prioritize children	2
	Bullying by superior/supervisor	1
	Children around make situation less stressful	1
	Risk group	1
Total		27
Campus life and infrastructure	Access to infrastructure	5
	Access to colleagues/research team/networking	6
	Daily routine/structure	3
	Parent guilt	1
Total		15
Other	Challenge not related to parenting	5
	Nothing	2
	Child with behavioral/education issues	1
	No answer	1
Total		9

## Footnotes

^1^Ph.D. candidate is the lowest career level we considered, followed by post-doctoral researcher. Lecturers/Adjunct faculty are full-time appointments but not usually on the tenure track although there are some exceptions. Post-docs often move into a position as assistant professor, then, once tenured, become associate professor, then full professor. We used the broad term “researcher” for those with a research appointment at a university who do not fall in these categories.

^2^Note that the rank of “lecturer” is interpreted differently for those in the USA-based system and those in the UK-based system.

^3^The term “lockdown” (and its related term “reopening”) is indexical to situations in respondents' own countries.

^4^Note that the 1 Black or African American respondent was added to the “other” group.
