# Supplementary material for: Challenges and Opportunities for Academic Parents During COVID-19
Source: Front Psychol. 2021 Aug 18;12:645734. doi: 10.3389/fpsyg.2021.645734 (PMC8416525; doi:10.3389/fpsyg.2021.645734)
Supplement: Supplementary file 1 [file Data_Sheet_1.docx]

# Supplementary data: Survey questions

Challenges and opportunities for academic parents during the 2020 COVID-19 pandemic: an international study

Start of Block: Informed Consent

Q1
Welcome to this questionnaire on "Academic parents during COVID-19"!

We (Eva Lantsoght, Kathleen Leemans, Misty Paig-Tran, Silvia Tavares, Yvonne Tse Crepaldi) are interested in understanding the lived experiences of academic parents during the COVID-19 pandemic and associated lockdowns. For this study, you will be asked to answer questions about your experience as an academic parent during the COVID-19 pandemic and associated lockdowns.

Your responses will be kept completely confidential. Your personal information will not be published as part of the dataset. All entries are anonymous. You will be assigned a code and no published information will lead to you being identified. E-mail addresses are only collected to send the report of this study to the participants.

You may be reminded of unpleasant events and emotions during the COVID-19 pandemic. Should you have unresolved traumatic experiences, please contact a licensed medical professional and consider not participating in this survey. If you feel anxious when reading/ any question, you have the option to not answer the question.

The study should take you around 20 minutes to complete. Your participation in this research is voluntary. You will not have to pay anything to participate and will not be paid either. You have the right to withdraw at any point during the study by contacting the PI, who will then proceed to delete your contribution. The Principal Investigator of this study can be contacted at Eva Lantsoght elantsoght@usfq.edu.ec

All participants will be informed on the outcome of the study by the end of 2021 by email, provided that you leave your contact information at the end of the questionnaire.

IRB approval has been obtained through Universidad San Francisco de Quito with study number 2020-056M.

By clicking the button below, you acknowledge:

Your participation in the study is voluntary. You are 18 years of age. You are aware that you may choose to terminate your participation at any time for any reason. You understand your participation in this study and understand the risks and benefits participating in this study.

- I consent, begin the study (1)
- I do not consent, I do not wish to participate (2)

| Page Break |  |
| --- | --- |

End of Block: Informed Consent

Start of Block: Demographics

Q18
In this part of the survey, we collect general data.


What is your gender?

- Male (1)
- Female (2)
- Other/Prefer not to say (3)

| 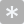 |
| --- |

Q13 What is your year of birth?

________________________________________________________________

| 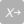 |
| --- |

Q8 What is your country of citizenship?

▼ Afghanistan (1) ... Zimbabwe (1357)

| 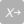 |
| --- |

Q4
In which country do you currently reside?

▼ Afghanistan (1) ... Zimbabwe (1357)

Q17 Choose one or more races that you consider yourself to be:

- White (1)
- Black or African American (2)
- American Indian or Alaska Native (3)
- Asian (4)
- Native Hawaiian or Pacific Islander (5)
- Latino/Hispanic (6)
- Other (7) ________________________________________________

Q16 What is your current position?

- PhD candidate (7)
- Researcher (9)
- Lecturer (6)
- Post-doctoral researcher (1)
- Assistant professor (2)
- Associate professor (3)
- Full professor (4)
- Other academic appointment (5)
- Other (10) ________________________________________________

Q66 Identify the FTE (full time equivalent, 1,0 fte = full-time appointment) of your position

|  | 0 | 0 | 0 | 0 | 0 | 1 | 1 | 1 | 1 | 1 | 1 | 1 | 1 | 1 | 1 | 1.5 |
| --- | --- | --- | --- | --- | --- | --- | --- | --- | --- | --- | --- | --- | --- | --- | --- | --- |

| FTE () | 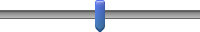 |
| --- | --- |

Q65 What is your field of study

- Agricultural and Biological Sciences (1)
- Arts and Humanities (2)
- Biochemistry, Genetics and Molecular Biology (3)
- Business, Management and Accounting (4)
- Chemical Engineering (5)
- Chemistry (6)
- Computer Science (7)
- Decision Sciences (8)
- Dentistry (9)
- Earth and Planetary Sciences (10)
- Economics, Econometrics and Finance (11)
- Energy (12)
- Engineering (13)
- Environmental Science (14)
- Health Professions (15)
- Immunology and Microbiology (16)
- Materials Science (17)
- Mathematics (18)
- Medicine (19)
- Multidisciplinary (20)
- Neuroscience (21)
- Nursing (22)
- Pharmacology, Toxicology and Pharmaceutics (23)
- Physics and Astronomy (24)
- Psychology (25)
- Social Sciences (26)
- Veterinary (27)

Q67 How many children currently live with you?

- 1 (9)
- 2 (10)
- 3 (11)
- 4 (12)
- 5 (13)
- 6 (14)
- more than 6 (15)

Q68 What are the ages of your children?

- Infant: 0 - 1 year (4)
- Toddler: 1 - 2 years (5)
- Preschooler: 3 - 6 years (6)
- Primary school: 7 - 12 years (7)
- Secondary school: 13 - 18 years (8)

Q69 What is your relationship status?

- Single (1)
- With a partner, living together (2)
- With a partner, living separately (4)
- Other (3) ________________________________________________

Q70 If you co-parent, how much FTE does your partner work?

|  | 0 | 0 | 0 | 0 | 0 | 1 | 1 | 1 | 1 | 1 | 1 | 1 | 1 | 1 | 1 | 1.5 |
| --- | --- | --- | --- | --- | --- | --- | --- | --- | --- | --- | --- | --- | --- | --- | --- | --- |

| FTE () | 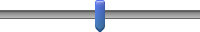 |
| --- | --- |

Q18  Please leave your email address here, if you want to receive a report with the outcome of this study.

________________________________________________________________

| Page Break |  |
| --- | --- |

End of Block: Demographics

Start of Block: Childcare

Q71 What was your childcare situation before the pandemic? Select all that apply.

- Daycare (1)
- Children in school (2)
- After-school care (3)
- Before-school care (4)
- Nanny (5)
- Au pair (6)
- Stay-at-home partner (7)
- Grandparents (8)
- Other relatives (9)
- Other (10) ________________________________________________
- No childcare (11)

Q72 What is your childcare situation during lockdown (or strongest restrictions) of pandemic? Select all that apply.

- Daycare (1)
- Children in school (2)
- After-school care (3)
- Before-school care (4)
- Nanny (5)
- Au pair (6)
- Stay-at-home partner (7)
- Grandparents (8)
- Other relatives (9)
- Other (10) ________________________________________________
- No childcare (11)

Q73 What is your childcare situation during (partial) release of restrictions of pandemic? Select all that apply.

- Daycare (1)
- Children in school (2)
- After-school care (3)
- Before-school care (4)
- Nanny (5)
- Au pair (6)
- Stay-at-home partner (7)
- Grandparents (8)
- Other relatives (9)
- Other (10) ________________________________________________
- No childcare (11)

Q74 Identify on a scale from 1-5 how satisfied you are with your childcare situation

|  | Extremely dissatisfied (25) | Dissatisfied (26) | Neither satisfied nor dissatisfied (27) | Satisfied (28) | Extremely satisfied (29) |
| --- | --- | --- | --- | --- | --- |
| Before the pandemic (1) |  |  |  |  |  |
| During lockdown of pandemic (2) |  |  |  |  |  |
| During partial release of measures of pandemic (3) |  |  |  |  |  |

Q75 Select all for whom you have additional care responsibilities during the pandemic

- Elderly relatives (1)
- Partner (2)
- Neighbors (3)
- Extended family (4)
- Other (5) ________________________________________________
- None (6)

Q76 Rate for the following statements how much you agree or disagree on a scale from 1-5:

|  | Strongly disagree (48) | Somewhat disagree (49) | Neither agree nor disagree (50) | Somewhat agree (51) | Strongly agree (52) |
| --- | --- | --- | --- | --- | --- |
| Arranging childcare during the pandemic is stressful (18) |  |  |  |  |  |
| I have taken on more childcare duties during the pandemic than before (19) |  |  |  |  |  |
| My co-parent has taken on more childcare duties during the pandemic than before (20) |  |  |  |  |  |
| I feel supported by my family as an academic parent (21) |  |  |  |  |  |
| I feel supported by my community as an academic parent (22) |  |  |  |  |  |
| I feel more involved in the education of my children as a result of the pandemic (30) |  |  |  |  |  |
| I am worried about sending my children to school or daycare during this pandemic (31) |  |  |  |  |  |

Q77
In this part, we would like you to answer open-ended questions regarding your childcare situation.


 Can you share your main challenges regarding childcare as an academic parent during COVID-19?

________________________________________________________________

________________________________________________________________

________________________________________________________________

________________________________________________________________

________________________________________________________________

Q78 What has worked well for you in terms of childcare during this pandemic?

________________________________________________________________

________________________________________________________________

________________________________________________________________

________________________________________________________________

________________________________________________________________

Q79 What are your best tips for working from home with limited childcare during the pandemic, if you’ve encountered this situation?

________________________________________________________________

________________________________________________________________

________________________________________________________________

________________________________________________________________

________________________________________________________________

Q81 How is your physical working space? What has worked well for you? What hasn't?

________________________________________________________________

________________________________________________________________

________________________________________________________________

________________________________________________________________

________________________________________________________________

Q82 How do you combine distance learning of your children with your academic work, if you’ve encountered this situation?

________________________________________________________________

________________________________________________________________

________________________________________________________________

________________________________________________________________

________________________________________________________________

Q83 Describe your favorite academic parenting moment from the COVID-19 pandemic and your least favorite one.

________________________________________________________________

________________________________________________________________

________________________________________________________________

________________________________________________________________

________________________________________________________________

End of Block: Childcare

Start of Block: Work as an academic parent

Q84 On a scale from 1 – 5 (very negatively – very positively), how has being an academic parent during the pandemic affected your research?

|  | Extremely negative (25) | Somewhat negative (26) | Neither positive nor negative (27) | Somewhat positive (28) | Extremely positive (29) |
| --- | --- | --- | --- | --- | --- |
| Research in general (1) |  |  |  |  |  |
| Data collection (2) |  |  |  |  |  |
| Lab work (3) |  |  |  |  |  |
| Analysis (4) |  |  |  |  |  |
| Reading (5) |  |  |  |  |  |
| Writing (6) |  |  |  |  |  |
| Dissemination activities (7) |  |  |  |  |  |

Q86 On a scale from 1 – 5 (very negatively – very positively), how has being an academic parent during the pandemic affected your teaching?

|  | Extremely negative (25) | Somewhat negative (26) | Neither positive nor negative (27) | Somewhat positive (28) | Extremely positive (29) |
| --- | --- | --- | --- | --- | --- |
| Teaching in general (1) |  |  |  |  |  |
| Class preparation (2) |  |  |  |  |  |
| Lectures (3) |  |  |  |  |  |
| Asynchronous activities (4) |  |  |  |  |  |
| Grading (5) |  |  |  |  |  |
| Contact with students (6) |  |  |  |  |  |
| Supervision of students (7) |  |  |  |  |  |

Q87 Rate for the following statements how much you agree or disagree on a scale from 1-5:

|  | Strongly disagree (20) | Somewhat disagree (21) | Neither agree nor disagree (22) | Somewhat agree (23) | Strongly agree (24) |
| --- | --- | --- | --- | --- | --- |
| It is harder for me to do my work duties as an academic parent than for my colleagues who are not parents (1) |  |  |  |  |  |
| I have managed to stay on top of my duties as an academic (2) |  |  |  |  |  |
| I have managed to stay on top of my duties as a parent (3) |  |  |  |  |  |
| I have considered withdrawing from my program, resign from my program, or go part-time to be able to provide more childcare (4) |  |  |  |  |  |
| I am worried about being able to financially provide for my family through my academic work (5) |  |  |  |  |  |

Q88
In this part, we would like you to answer open-ended questions regarding work as an academic parent.


 Which challenges have you encountered in your research during the COVID-19 pandemic? How did being a parent relate to these challenges?

________________________________________________________________

________________________________________________________________

________________________________________________________________

________________________________________________________________

________________________________________________________________

Q90 Which opportunities have you encountered in your research during the COVID-19 pandemic? How did being a parent relate to these opportunities?

________________________________________________________________

________________________________________________________________

________________________________________________________________

________________________________________________________________

________________________________________________________________

Q91  How has the increased use of digital tools and cloud environments influenced your academic work? Discuss any advantages or disadvantages to the increased use of technology in relation to your parenting.

________________________________________________________________

________________________________________________________________

________________________________________________________________

________________________________________________________________

________________________________________________________________

Q92 Discuss any work-related financial uncertainties you are facing, and how this situation could affect your family budget.

________________________________________________________________

________________________________________________________________

________________________________________________________________

________________________________________________________________

________________________________________________________________

Q93 Discuss how the pandemic has changed work-related travel for you, and the advantages and disadvantages of this situation.

________________________________________________________________

________________________________________________________________

________________________________________________________________

________________________________________________________________

________________________________________________________________

End of Block: Work as an academic parent

Start of Block: Overall time availability and work-life balance:

| 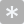 |
| --- |

Q94 Estimate how you spent your time per week (168 hours) before the pandemic:

- Work : _______ (1)
- Sleep : _______ (2)
- Commute : _______ (3)
- Childcare : _______ (4)
- Household : _______ (5)
- Other care activities : _______ (6)
- Leisure : _______ (7)
- Other : _______ (8)

Total : ________

| 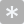 |
| --- |

Q95 Estimate how you spent your time per week (168 hours) during the pandemic:

- Work : _______ (1)
- Sleep : _______ (2)
- Commute : _______ (3)
- Childcare : _______ (4)
- Household : _______ (5)
- Other care activities : _______ (6)
- Leisure : _______ (7)
- Other : _______ (8)

Total : ________

Q96 Rate for the following statements how much you agree or disagree on a scale from 1 - 5:

|  | Strongly disagree (18) | Somewhat disagree (19) | Neither agree nor disagree (20) | Somewhat agree (21) | Strongly agree (22) |
| --- | --- | --- | --- | --- | --- |
| Being an academic parent has reduced my time availability for work duties during the pandemic (1) |  |  |  |  |  |
| Being an academic parent has reduced my time availability for volunteering duties such as peer review during the pandemic (2) |  |  |  |  |  |
| Being an academic parent has reduced my publication output during the pandemic (3) |  |  |  |  |  |
| I have found a better work-life balance as an academic parent during the pandemic (4) |  |  |  |  |  |
| My work hours are more flexible during the pandemic than before (5) |  |  |  |  |  |

End of Block: Overall time availability and work-life balance:

Start of Block: University support

Q97 Rate for the following statements how much you agree or disagree on a scale from 1 - 5:

|  | Strongly disagree (18) | Somewhat disagree (19) | Neither agree nor disagree (20) | Somewhat agree (21) | Strongly agree (22) |
| --- | --- | --- | --- | --- | --- |
| I feel supported by my university as an academic parent during the pandemic. (1) |  |  |  |  |  |
| My university has actively helped me achieving balance between work and childcare duties during the pandemic (2) |  |  |  |  |  |
| My university has actively helped me find childcare alternatives (3) |  |  |  |  |  |
| My university has a welcoming attitude to parents (4) |  |  |  |  |  |
| My colleagues understand the challenges of being an academic parent (5) |  |  |  |  |  |
| My superiors understand the challenges of being an academic parent (6) |  |  |  |  |  |

Q98
In this part, we would like you to answer open-ended questions regarding university support.


 What has your university done to support academic parents? What has worked for your situation and what did not work?

________________________________________________________________

________________________________________________________________

________________________________________________________________

________________________________________________________________

________________________________________________________________

Q99  If you are on a fixed-term contract with requirements to fulfil before you can make the next step in your career (eg. tenure clock), discuss if and how your university has accounted for the current pandemic, and if these measures are different or the same for academics with children.

________________________________________________________________

________________________________________________________________

________________________________________________________________

________________________________________________________________

________________________________________________________________

End of Block: University support

Start of Block: Thank you

Q100
Thank you very much for participating in this study!


 Do you wish to receive a report of the results of the study? If your answer is Yes, we will keep your contact information. If your answer is No, your contact information will be removed.

- Yes (5)
- No (6)

End of Block: Thank you
